# Bacterial metabolic remodeling by convergent evolution unlocks nutrient availability after a host switch

**DOI:** 10.1126/sciadv.adw9419

**Published:** 2026-02-06

**Authors:** Amy C. Pickering, Jamie Gorzynski, Grace Taylor-Joyce, Rhodri Evans, Willow Fox, Pedro Melo, Joana Alves, Hannah Schlauch, Fiona Sargison, Gonzalo Yebra, Natalie Ring, J. Ross Fitzgerald

**Affiliations:** The Roslin Institute and Edinburgh Infectious Diseases, University of Edinburgh, Easter Bush, Midlothian, Scotland, UK.

## Abstract

New pathogens typically arise from host jump events between species. *Staphylococcus aureus* is a multihost pathogen responsible for a global burden of human disease and a leading cause of intramammary infection in dairy cattle. Here, we demonstrate that following historical human-to-bovine host switch events, *S. aureus* has undergone adaptive metabolic remodeling in response to distinct nutrient availability in the dairy niche. In particular, we found that bovine *S. aureus* has evolved the capacity for protease-mediated degradation of casein, a protein abundant in bovine milk, to access nutrients for proliferation. This phenotype has evolved convergently in different *S. aureus* lineages via mutations in distinct gene loci driving overexpression of the protease aureolysin. Together, we have dissected a key host-adaptive trait, which facilitates the enzymatic release of nutrients from a substrate specific to the new host milieu. These findings highlight the remarkable evolutionary plasticity of a major bacterial pathogen underpinning its multihost species tropism.

## Teaser

*Staphylococcus aureus* has undergone metabolic remodeling to adapt to the dairy niche.

## INTRODUCTION

The emergence of new pathogens often occurs after host-switching events between species, underpinned by adaptation to the new ecological niche. However, our understanding of the evolutionary processes and mechanisms driving bacterial host adaptation remains limited. In particular, the capacity to pivot to distinct nutrient availability in the new niche is essential for bacterial proliferation and critical for a successful host switch (*1*). Specialist bacteria have been reported to undergo genome reduction to specialize for specific nutrients available in a single niche (*1*–*4*). In contrast, generalist bacteria may be required to maintain a broad range of metabolic pathways at their disposal for deployment simultaneously or sequentially (*5*). There is increasing awareness of the complexities of nutrient availability for bacteria in different niches (*6*–*8*) and the role of nutritional immunity in limiting bacterial infection (*9*). However, the ability of multihost pathogens such as *Staphylococcus aureus* to pivot to distinct nutrients after a host switch is not well understood.

Human domestication of cattle for milk and associated dairy products provided a major niche for the expansion of bacteria, and evidence for dairy niche adaptation has been reported (*10*). For example, *Lactococcus lactis*, used widely for production of cheese and buttermilk, exhibits enhanced lactose utilization via plasmid-encoded lactose metabolism (*11*, *12*), while the bovine intramammary pathogen *Streptococcus agalactiae* has acquired bovine-specific lactose and fructose operons (*13*, *14*). Furthermore, enhanced utilization of casein, the major protein constituent of milk, is a feature of *L. lactis* (*11*) and mastitis isolates of *Escherichia coli* (*15*).

*S. aureus* is a leading cause of bovine mastitis in the global dairy industry, and all contemporary lineages can be traced to a small number of independent host-switching events from humans which were followed by adaptation and clonal expansion (*16*, *17*). A combination of gene acquisition, loss, and diversification has been reported to be associated with adaptation to the bovine niche including mobile genetic elements (MGE) encoding host-specific effectors of virulence and immune evasion (*17*–*20*). Bovine *S. aureus* strains also exhibit enhanced lactose utilization compared to strains causing disease in other host species (*21*, *22*). However, the functional evolutionary basis for *S. aureus* adaptation to the nutrients available in the dairy niche is poorly understood. Here, we have used a multiomics approach to investigating the ability of *S. aureus* to successfully transition to a new ecological niche in the face of distinct nutrient availability. We found that bovine *S. aureus* has undergone metabolic remodeling since the host jump and that enhanced expression of the protease aureolysin is key to unlocking the nutrient potential of milk. This adaptive phenotype has evolved convergently in different *S. aureus* lineages via mutations in distinct gene loci, highlighting the remarkable capacity of *S. aureus* to expand into new host niches.

## RESULTS

### Bovine *S. aureus* lineages exhibit a clotting phenotype associated with enhanced growth in milk

**Fig. 1.**
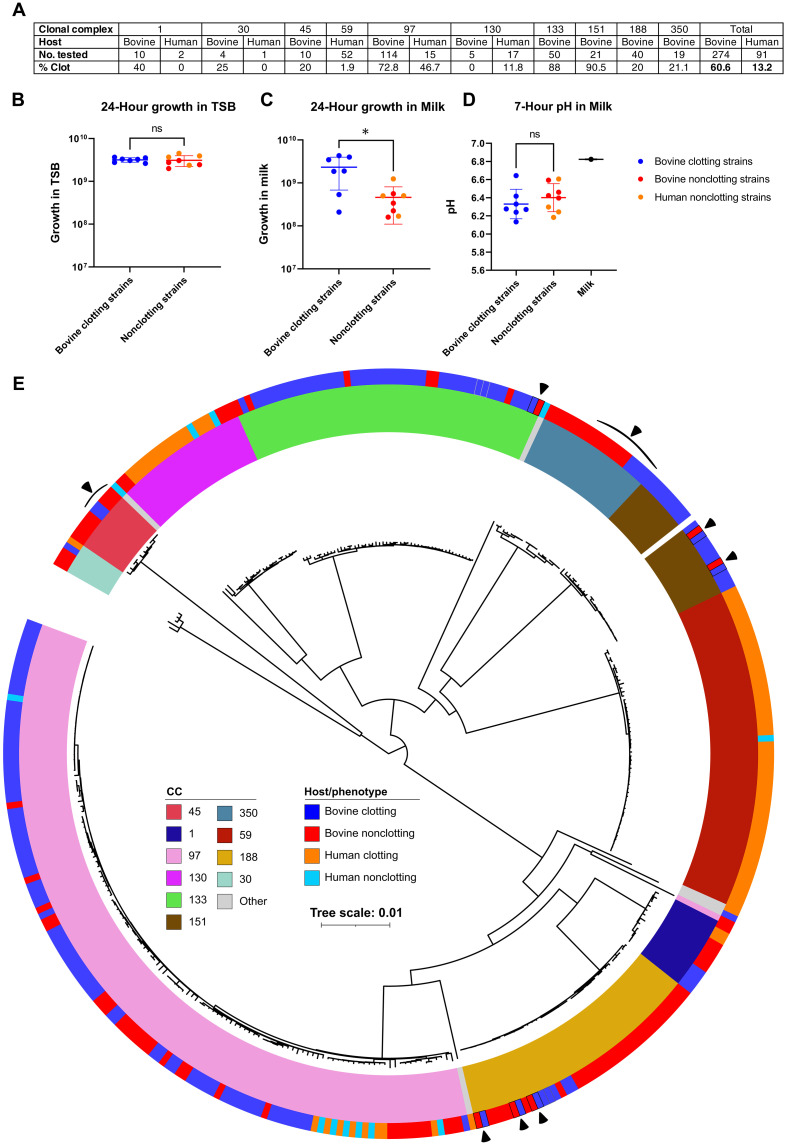
Bovine strains from multiple lineages have evolved a milk clotting phenotype associated with enhanced growth in milk. (**A**) Frequency of the milk clotting phenotype in the isolates tested. (**B** and **C**) Colony-forming unit (CFU) analysis of a diverse selection of bovine clotting, bovine nonclotting, and human nonclotting strains after 24-hour growth in (B) TSB (two-sided Welch’s *t* test, *t* = 0.2320, df = 9.838, *P* = 0.8213) and (C) milk (two-sided Welch’s *t* test, *t* = 2.945, df = 6.482, *P* = 0.0235). (**D**) pH analysis of the same strains after milk clotting was completed at 7 hours postinoculation (two-sided *t* test, *t* = 0.8601, df = 13, *P* = 0.4053). Asterisks indicate the level of significance: *P* > 0.05 [not significant (ns)]; 0.01 < *P* ≤ 0.05 (*). Each data point represents *n* = 3 biological replicates for individual strains. Error bars, means ± SD. (**E**) A midpoint-rooted maximum-likelihood phylogeny of core SNPs across 365 isolates that were tested for clotting, corrected for ascertainment bias. Variants were called against a reference genome for the RF122 strain (AJ938182). Inner ring colors are used to indicate the CC of isolates, and outer ring colors indicate the milk clotting phenotype. Branches are drawn to the scale shown and measure the average number of substitutions per site. Black arrowheads are used to indicate pairs or clades of isolates with contrasting phenotypes that were analyzed in more detail, as referenced in the main text.

Previously, we identified that bovine *S. aureus* had increased capacity to use lactose (the major carbohydrate present in milk), in comparison to strains of human origin (*21*). To investigate this observation further, we compared the growth phenotype of bovine and human *S. aureus* in bovine milk. Using ex vivo milk infection experiments, we assessed 274 bovine and 91 human *S. aureus* strains from an array of common lineages [clonal complexes (CCs)] (table S1). Unexpectedly, 60.6% of bovine strains produced a clotting phenotype (separation into solid curds and liquid whey fractions) after 4 to 6 hours of incubation at 37°C, in comparison to 13.2% of human strains (Fig. 1A). Of note, the frequency of milk clotting varied in different bovine CCs with those that are globally distributed and long-established in ruminants e.g., CC97, CC133, and CC151 [17], exhibiting highest clotting frequencies among bovine strains, of 72.8, 88, and 90.5%, respectively (Fig. 1A). In comparison, those CCs more recently associated with cattle, e.g., CC1, CC30, and CC188, tended to have lower frequencies of milk clotting among bovine strains with 40, 25, and 20%, respectively (Fig. 1A).

To investigate the potential relevance of the milk clotting phenotype for bacterial fitness, seven selected bovine clotting strains (1xCC97, 3xCC133, 2xCC151, and 1xCC350), four bovine nonclotting strains (2xCC1, 1xCC97, and 1xCC350), and four human nonclotting strains (2xCC97 and 2xCC130) were cultured in both milk and tryptone soya broth (TSB) at 37°C. There were no differences in bacterial growth rate or yield of strains cultured in TSB (Fig. 1B). However, bovine strains that clotted milk demonstrated an enhanced growth yield in milk compared to nonclotting strains of both bovine and human origin (Fig. 1C). *S. aureus*–induced milk clotting is not due to milk acidification (curdling) as there was no significant reduction in pH of the milk media by the time clotting had occurred (Fig. 1D). Overall, we have identified a milk clotting phenotype that is associated with enhanced growth and produced at high frequency by different global bovine *S. aureus* lineages.

### Phylogenetic analysis demonstrates a paraphyletic distribution of the milk clotting phenotype

To investigate the distribution and evolutionary origin of the milk clotting phenotype in *S. aureus*, we performed a maximum-likelihood phylogenetic analysis of genomes from 365 *S. aureus* strains from bovine and human origin, representing 10 lineages from 22 countries (table S1). The phylogeny was strongly delineated by CC, with most CCs demonstrating a paraphyletic distribution of the milk clotting phenotype (Fig. 1E). For the three bovine lineages with the highest frequency of milk clotting (CC97, CC133, and CC151), the phylogeny indicates that the most recent common ancestor of each CC had the ability to clot milk, with sporadic loss-of-phenotype events occurring throughout the evolutionary history of each lineage (Fig. 1E). In contrast, for bovine lineages with lower frequencies of milk clotting (CC1, CC30, CC45, CC188, and CC350), the phylogeny indicates sporadic gain-of-phenotype events (Fig. 1E). These data suggest that minimal genetic variations might be sufficient to confer or lose the milk clotting phenotype.

### Transcriptomic analysis of bovine *S. aureus* during growth in milk reveals adaptive metabolic remodeling

To investigate the basis for the enhanced growth phenotype in milk, we carried out RNA sequencing of 11 *S. aureus* strains from the CC97 global lineage including bovine clotting (*n* = 4), bovine nonclotting (*n* = 3), and human nonclotting strains (*n* = 4) in the early (2 hours) stages of growth in 50% milk-TSB media, in comparison to growth in TSB only. Strains were selected according to their distribution across the CC97 lineage, clotting, growth phenotype, and host species origin (fig. S1, A to C).

**Fig. 2.**
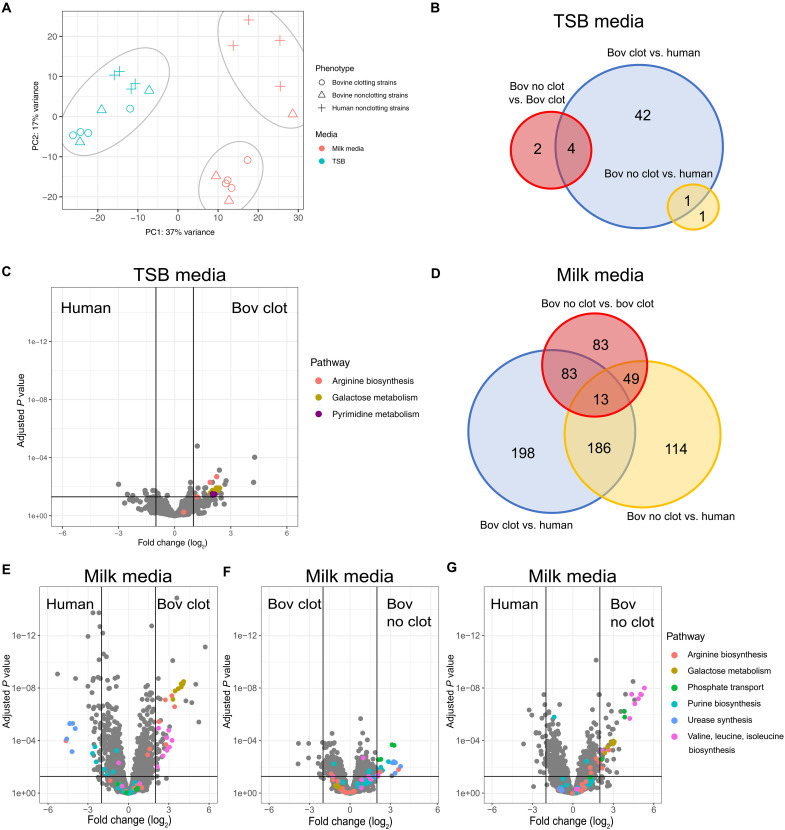
Transcriptomic analysis demonstrates metabolic differences between clotting and nonclotting strains in milk. (**A**) Principal component (PC) and K-means clustering analysis, each separate cluster is circled in gray. (**B**) Venn diagram showing differentially expressed genes in TSB. (**C**) Volcano plot demonstrating the metabolic differences between bovine clotting and nonclotting strains in TSB. (**D**) Venn diagram showing differentially expressed genes in 50% milk/TSB media between experimental groups. (**E**) Volcano plot demonstrating the metabolic differences between bovine clotting and nonclotting strains in 50% milk/TSB. (**F**) Volcano plot demonstrating the metabolic differences between bovine clotting and human nonclotting strains in 50% milk/TSB. (**G**) Volcano plot demonstrating the metabolic differences between bovine nonclotting and human nonclotting strains in 50% milk/TSB. Differential expression was determined as *P*_adj_ of ≤0.05 and log_2_ fold change of ≤−1, ≥1 for (B) to (D) and log_2_ fold change of ≤−2, ≥2 for (E) to (G).

Principal component and K-means clustering of global transcriptomic data revealed that bovine and human strains clustered together when cultured in nutrient-rich media (TSB) but formed two separate clusters when grown in milk; one cluster containing all bar one of the bovine strains with the second cluster containing all human strains and a single outlier bovine strain (Fig. 2A). In TSB, only 51 genes were differentially expressed (*P* adjusted value of ≤0.05 and log_2_ fold change of ≤−1, ≥1) between experimental groups, with the majority more highly expressed in the bovine clotting strains compared to the human nonclotting strains, including seven genes involved in lactose metabolism (*lacABCDFEG*), four genes involved in arginine biosynthesis (*argB*, *argC*, *argH*, and *argJ*), and four genes involved in pyrimidine metabolism (*carB*, *pyrAA*, *pyrE*, and *pyrF*) (Fig. 2, B and C, and table S2). Notably, when cultured in milk, a total of 726 genes were differentially expressed (*P* adjusted value of ≤0.05 and log_2_ fold change of ≤−1, ≥1) between experimental groups (Fig. 2D). We elected to focus on the 148 genes in milk that were differentially expressed with a log_2_ fold change of ≤−2, ≥2 for further analysis (table S3). Of the 89 genes annotated to Kyoto Encyclopedia of Genes and Genomes pathways, 71.9% are predicted to have a role in metabolism. All bovine strains, irrespective of milk clotting phenotype, demonstrated elevated expression of valine, leucine, and isoleucine [branched chain amino acids (BCAA)] biosynthesis, arginine biosynthesis, and galactose metabolism in comparison to human strains (Fig. 2, E and G, and figs. S2B and S3B). Down-regulation of expression of urease and purine metabolism genes was observed in bovine clotting strains compared to nonclotting bovine and human strains (Fig. 2, E and F, and figs. S2 and S3), while bovine nonclotting strains exhibited increased phosphate transport expression (*pstSCAB*) associated with nucleotide biosynthesis (Fig. 2, F and G) (*23*). Last, we found that *S. aureus* clotting strains exhibited an elevated expression of genes encoding extracellular proteases in both milk and TSB in comparison to nonclotting strains from both cattle and humans (tables S2 and S3). Overall, these data infer that bovine *S. aureus* has undergone adaptation to facilitate increased utilization of lactose, to promote the synthesis of BCAAs and arginine, and increase the expression of extracellular proteases. We hypothesize that the metabolic remodeling observed has contributed to enhanced growth of *S. aureus* in the dairy niche.

### The milk clotting phenotype is dependent on the protease aureolysin

**Fig. 3.**
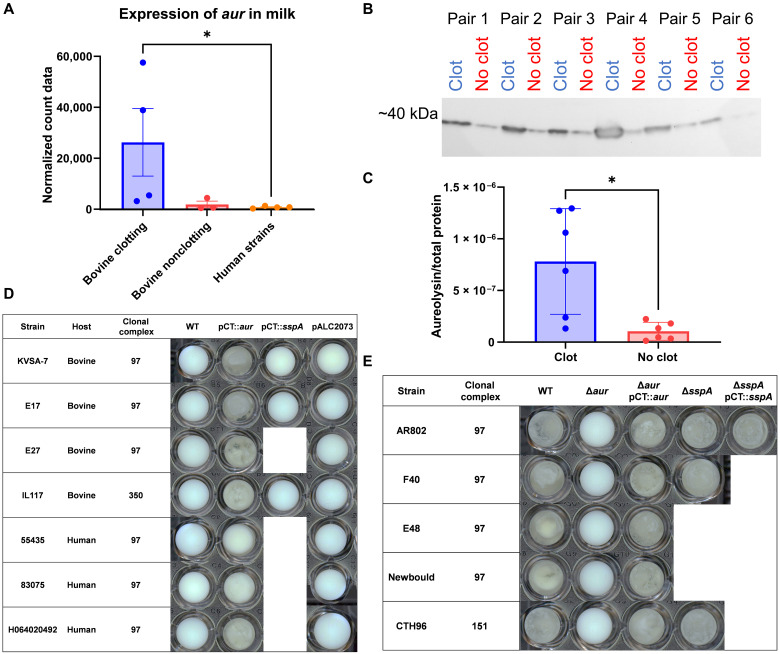
Increased expression of aureolysin in milk is required for milk clotting. (**A**) Normalized count data of Aur expression after 2 hours of incubation in 50% milk/TSB for each experimental group (Kruskal-Wallis statistic = 6.053, *P* = 0.0348). (**B**) Western blot analysis of concentrated supernatant from paired isolates probed with anti-Aur IgY. (**C**) Normalized Aur expression in relation to Revert Total Protein Stain. Each data point represents individual supernatant samples (*t* test, *P* = 0.0137). (**D**) Representative milk clotting phenotype of strains capable of clotting milk, deletion mutants, and complemented strains after 24 hours at 37°C under static conditions in a 96-well plate. (**E**) Representative milk clotting phenotype of strains not capable of clotting milk and complemented strains after 24 hours at 37°C under static conditions in a 96-well plate. Each data point represents individual strains. Error bars, means ± SEM. WT, wild type.

Of note, the protease aureolysin (Aur) gene (*aur*) exhibited 14.5-fold increased expression in bovine clotting strains compared to bovine nonclotting strains and 37-fold increased expression compared to human nonclotting strains during growth in milk (Fig. 3A). The strong correlation between the clotting phenotype and *aur* gene expression led us to investigate Aur protein expression levels using bespoke Aur-specific antibodies (Eurogentec). From the *S. aureus* phylogeny, six pairs of closely related strains [<100 single-nucleotide polymorphism (SNPs)] with opposing milk clotting phenotypes were examined (Fig. 1). Notably, for each pair, Aur protein expression was elevated in the clotting strain compared to the related nonclotting strain (Fig. 3, B and C).

To determine whether this enhanced expression is responsible for milk clotting, we initially complemented a mutant derivative [USA300Δprotease (*24*)] of USA300 *S. aureus* strain (LAC) deficient in expression of all known extracellular proteases, with the *aur* gene and its native promoter cloned into pCT [a derivative of pALC2073 (*25*)]. USA300Δprotease pCT::*aur* mediated clotting of milk after 2 hours of induction, whereas USA300Δprotease and USA300Δprotease pALC2073 (empty vector) were unable to clot milk after 24 hours (fig. S4). Furthermore, 100 μg of the concentrated supernatant from USA300Δprotease pCT::*aur* was sufficient to clot 5 ml of milk within 10 min. These data indicate that overexpression of Aur in the supernatant of an extracellular protease-deficient strain background is sufficient to mediate milk clotting. This was further examined through the overexpression of Aur via pCT::*aur* in six CC97 nonclotting strains of bovine origin, three of human origin, and one CC350 strain, each of which was sufficient to promote milk clotting (Fig. 3D).

To determine whether Aur is essential for the milk clotting phenotype, *aur* deletion mutant strains were generated in bovine clotting strains, including the archetypal bovine strain Newbould (ST115), three additional CC97 strains, and the CC151 strain CTH96. Of the five Aur-deficient strains, four were unable to clot milk after 24 hours at 37°C, and the other was significantly delayed (Fig. 3E). The clotting phenotype was restored by complementation with pCT::*aur* (Fig. 3E). As Aur is known to trigger a proteolytic cascade and is required for the activation of SspA, which subsequently activates SspB (*26*), we considered that the ablation of the clotting phenotype in the *aur* deletion mutant could be explained by a lack of SspA activation by Aur. Accordingly, deletion mutants of *sspA* were generated in three strains (AR802 and F40 from CC97 and CTH96 from CC151). All three *sspA* mutants clotted milk in a manner similar to the wild-type strains, confirming that Aur is essential for the milk clotting phenotype, and this is independent of SspA (Fig. 3E). Last, overexpression by complementation (fig. S5) with pCT::*sspA* was not sufficient to induce milk clotting by any strain (Fig. 3, D and E). Together, these data indicate that enhanced Aur expression by *S. aureus* is sufficient and required to mediate the milk clotting phenotype and is independent of downstream proteases.

### Aureolysin mediates digestion of casein, milk clotting and is required for enhanced growth of bovine *S. aureus* strains in milk

**Fig. 4.**
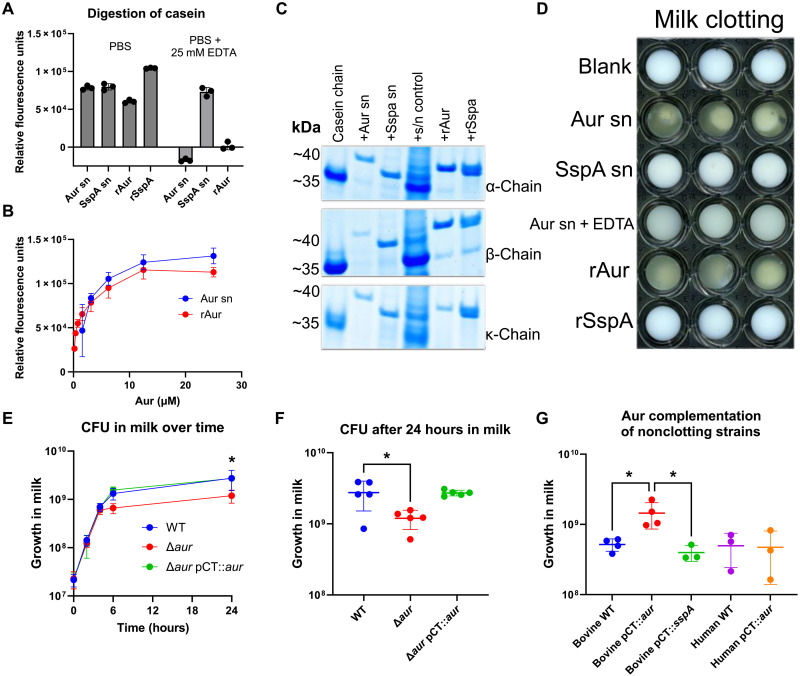
Aureolysin digests casein, mediates milk clotting, and promotes enhanced growth in milk. (**A**) FTC-casein digestion by 1 μM concentrated supernatant (sn) or recombinant proteins in the presence or absence of 25 mM EDTA as measured by FRET. (**B**) Dose-dependent digestion of FTC-casein by Aur present in the supernatant of USA300Δprotease pCT::*aur* or purified Aur as measured by FRET. Each data point represents *n* = 3 replicates. Error bars, means ± SD. (**C**) Digestion of individual chains of casein after 1 hour 30 min of incubation at room temperature as evaluated by SDS–polyacrylamide gel electrophoresis (SDS-PAGE). (**D**) Milk clotting of 1 μM concentrated supernatant (sn) or recombinant proteins after incubation at 37°C with shaking for 24 hours. (**E**) CFU analysis over time in milk of bovine clotting strains, corresponding *aur* deletion mutants, and complemented Aur strains. (**F**) Growth at 24 hours of the same strains used in (E) [matched one-way analysis of variance (ANOVA) with Dunnet’s multiple comparisons test, *F* = 11.42, adjusted *P* = 0.0293]. (**G**) CFU analysis of bovine and human nonclotting strains compared to those complemented with either Aur or SspA after 24-hour growth in milk (one-way ANOVA with Tukey’s multiple comparisons test, *F* = 6.076, *P* = 0.0065). Each data point represents *n* = 3 biological replicates for individual strains. Error bars, means ± SD.

Our transcriptomic and experimental data suggest that bovine strains have adapted to the ecological niche of the udder through metabolic remodeling and enhanced Aur expression. As casein digestion has been reported to promote milk clotting (*27*), we considered that Aur-dependent milk clotting may be mediated by casein digestion. To test this hypothesis, we examined the digestion of fluorescently labeled casein using fluorescence resonance energy transfer (FRET) (Pierce). As SspA is known to digest casein (*28*), it was used as a positive control. Both concentrated supernatant samples (USA300Δprotease pCT::*aur* and KVSA-7 pCT::*sspA*) purified Aur, and recombinant SspA (fig. S6) mediated dose-dependent FTC-casein digestion (Fig. 4, A and B) and degradation of all three chains of casein (Fig. 4C). However, only purified Aur or the concentrated supernatant of USA300Δprotease pCT::*aur* induced milk clotting, a phenotype that could be inhibited by 25 mM EDTA (Fig. 4D).

Next, we tested whether Aur is required for enhanced growth in milk. Comparison of bovine *S. aureus* clotting wild type, Δ*aur*, and pCT::*aur*-complemented strains demonstrated that loss of Aur expression resulted in reduced growth after 24 hours in milk (Fig. 4, E and F). Similarly, overexpression of Aur but not SspA in nonclotting bovine strains (3xCC97 and 1xCC350) enhanced growth in milk (Fig. 4G). In contrast, for human nonclotting strains (3xCC97), overexpression of Aur mediated clotting but did not result in enhanced growth (Fig. 4G). These data are consistent with our global transcriptomic analysis that revealed metabolic remodeling by bovine *S. aureus* compared to human strains, which facilitates the Aur-dependent enhanced growth observed in milk. Together, these data provide evidence for the functional adaptive evolution of bovine *S. aureus* to the dairy niche since the host switch from humans.

### Genome-wide association analysis reveals mutations associated with sporadic loss of clotting in bovine *S. aureus* populations

**Fig. 5.**
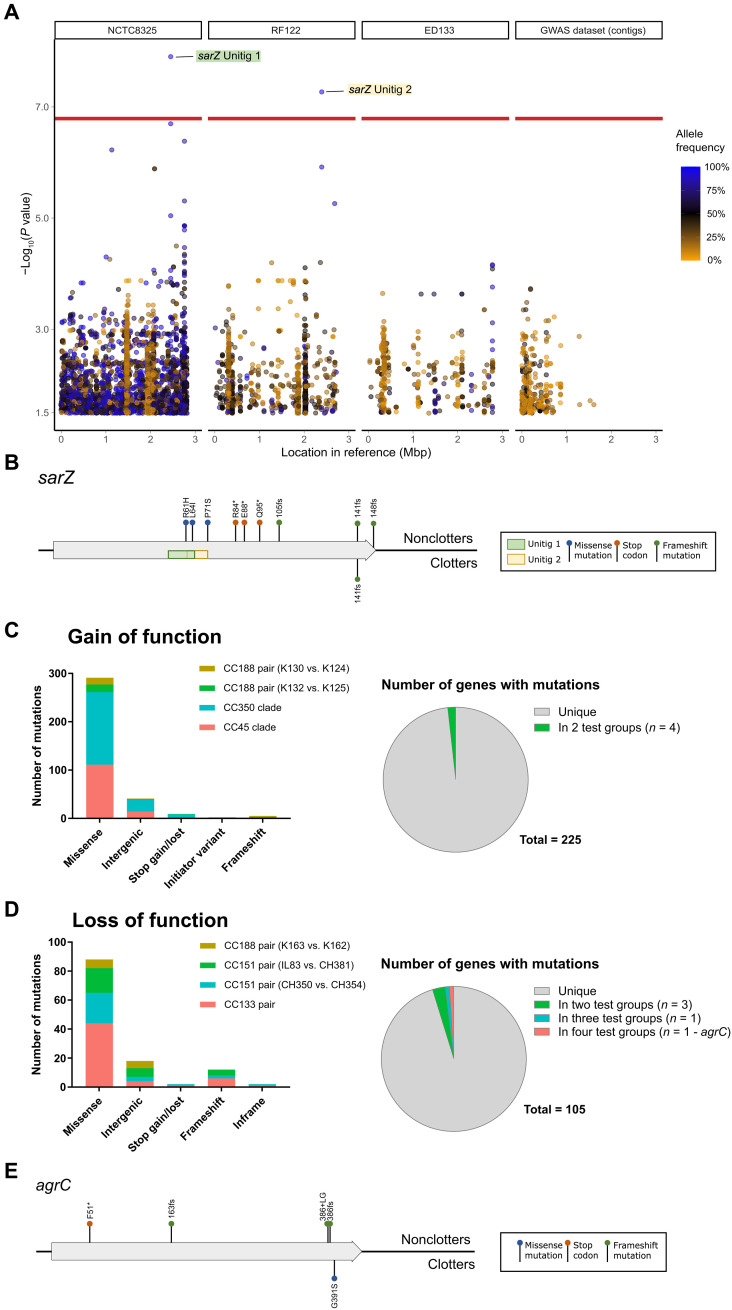
Distribution of SNPs and unitig features associated with gain or loss of the milk clotting phenotype. (**A**) Manhattan plot showing the significance of association (*P* < 10^−1.5^) with the milk clotting phenotype across the set of unitigs that constitute 254 genome sequences of isolates tested for clotting. The *X* axis shows the coordinates of each unitig motif in a reference genome containing that motif [in mega–base pairs (Mbp)], mapped iteratively as described in Materials and Methods. The *Y* axis shows the absolute magnitude of *P* values for each associated variant. Each point represents a single unitig and is colored proportionally to its frequency across the data, as indicated by the scale bar to the right of the panel. A horizontal red line shows the Bonferroni-corrected significance threshold used (α = 0.05). Unitigs that were significantly associated with the phenotype at this threshold are labeled to show the gene in which they were mapped, colored to match their location in (D). (**B** and **E**) Gene maps showing the location of SNPs identified in *sarZ* (B) or *agrC* (E) across all 254 sequences analyzed in the GWAS and paired SNP analyses, for which there is an expected negative impact in gene function. SNP locations are colored according to the type of mutation as indicated by the legend, along with the locations of significant unitigs in the *sarZ* gene, consistent with (A). Genes are depicted by gray arrows oriented from their start codon, with SNPs marked above or below the gene to indicate whether they occurred in clotters, nonclotters, or both. Asterisks indicate premature stop codons and fs the start of a frameshift mutation (**C** and **D**) The overall numbers of nonsynonymous SNPs identified in the paired SNP analysis for both gain-of-function (GOF) (C) and LOF (D) pairs. Pie charts show the number of genes containing these SNPs, colored to indicate the number of different pairs that contained SNPs in the same genes.

Having established the requirement for increased Aur expression in milk clotting and the enhanced growth phenotype of *S. aureus* across multiple bovine-associated lineages, we wanted to examine the evolutionary genetic basis of this phenotype. We performed two genome-wide association studies (GWAS) of clotting versus nonclotting isolates, one using 254 high-quality genomes spanning each of the major bovine lineages as described in Materials and Methods (Fig. 1E) and another including 104 high-quality genomes from the most abundant CC97 lineage only, to limit the effects of population structure on false positives. There were no significant unitigs identified in the CC97-specific GWAS (fig. S7). However, GWAS analysis of the larger dataset revealed two significant unitigs that mapped to the DNA binding site of *sarZ* a transcriptional promoter of *agr* expression (Fig. 5A and table S4) (*29*). Although most strains regardless of the milk clotting phenotype contain both unitig sequences (table S4), *sarZ* sequence alignment revealed putative loss-of-function (LOF) mutations in some nonclotting strains. Specifically, this included missense variants of the *sarZ* DNA binding site in ST151 strains, a nonsense mutation resulting in a premature stop codon in ST97, and an additional four unique genetic events leading to truncated *sarZ* genes in 10 nonclotting strains (7 from ST188, 2 from ST97, and 1 from ST130) (Fig. 5B). As *sarZ* is a positive regulator of the *agr* system (*29*), which in turn controls aureolysin expression (*30*), these function-altering SNPs may indirectly result in loss of the clotting phenotype. Of note, the GWAS did not reveal any mutations associated with gain of the clotting phenotype, although we highlight the possibility that the GWAS is underpowered to identify such traits with the dataset available.

### Comparative genomic analysis of the evolutionary genetic basis of the clotting phenotype

To further investigate the genetic basis for the clotting phenotype, we used a bespoke bioinformatic pipeline (described in detail in Materials and Methods). Briefly, we identified the genetic variation associated with four gain-of-function (GOF) events including two closely related GOF pairs of isolates in CC188 and two GOF clades in CC45 and CC350 (fig. S8A). In addition, we examined four pairs of isolates from three distinct lineages (CC133, CC151, and CC188) that demonstrated a loss-of-function (LOF) phenotype (fig. S8B). The genetic variation identified was compared to control strain sets that did not differ in clotting phenotype but had comparable SNP distances (table S4).

For the GOF analysis, we identified differences in gene content between nonclotting and clotting strains including variation in the complement of MGE with one of the CC188 pairs differing by a variant Sa3 phage (table S4). Furthermore, the CC188 pairs had differences in insertion sequence elements. To determine precisely the integration sites of the MGE, we carried out Nanopore long-read sequencing, which revealed a rearrangement and gene deletion event at one end of the Sa3 phage in the CC188 pair leading to deletion of the *lukH*, *lukG*, and *dapE* genes with the insertion of *scn* (fig. S9A) and a 192-kb inversion event that had occurred in strain K125 (CC188) that did not lead to disruption of neighboring genes (fig. S9A). In the same pair, there were differences in the complement of IS256 family transposons including one that disrupts a gene encoding a hypothetical protein that is adjacent to the edge of the inversion site identified in the nonclotting strain. The second IS256 family transposon does not disrupt any neighboring genes but is adjacent to a β-converting phage present in both strains. There is no apparent functional link with any of these gene differences and the clotting phenotype, although this cannot be ruled out categorically. However, there were no gene differences in common between the four GOF events examined (table S4).

For the SNP analysis of the GOF events (filtered as described in Materials and Methods), a total of 348 SNPs was identified that affect up to 225 genes, including 41 intergenic SNPs located within 100 bp of the coding sequence (CDS) start codon. There were 17 to 19 SNPs per pair (8 to 16 in controls) and 127 to 185 SNPs per clade (154 to 192 in controls). For the 307 SNPs that occurred within CDSs, 5 resulted in frameshifts, 9 resulted in a gained or lost stop codon, and the remaining 293 were missense mutations that altered one or more amino acids or the initiator codons (Fig. 5C and table S4). Of note, there were no SNPs identified in common between all four GOF events, indicating that distinct mutations were responsible for the clotting phenotype in different lineages (Fig. 5B). Further, only four genes with mutations were identified to be associated with more than one GOF event including *gltA*, *mutS*, and two encoding hypothetical proteins (Fig. 5B). No gene with mutations was found in more than two GOF events. One nonclotting strain contained a frameshift mutation in *agrA* that would result in a nonfunctional *agr* system and attenuated aureolysin expression. In another GOF event, the clotting strain encodes a frameshift mutation at nucleotide position 363 of *sigB*, which would be predicted to enhance *agr* function and promote aureolysin expression (table S4). Overall, this GOF analysis, in agreement with the GWAS, did not identify specific genes or SNPs that could be universally responsible for the clotting phenotype. Instead, different genes or SNPs underpinned the emergence of the milk clotting phenotype in different lineages in parallel consistent with convergent evolution.

### Loss of clotting in bovine *S. aureus* strains is associated with attenuation of *agr*

**Fig. 6.**
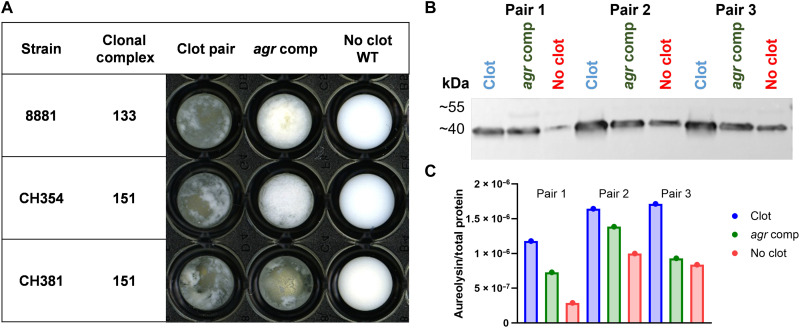
Agr complementation promotes the milk clotting phenotype through enhanced Aur expression. (**A**) Representative milk clotting phenotype of strains after 24 hours at 37°C with shaking in a 96-well plate. (**B**) Representative Western blot analysis of concentrated supernatant from paired isolates and complemented strains (*agr* comp) probed with anti-Aur IgY. (**C**) Normalized Aur expression in relation to Revert Total Protein Stain for the blot in (B). Biological triplicate concentrated supernatant samples were analyzed by Western blot and normalized with similar results.

For the four LOF events, comparison of gene content revealed differences in phage content (Sa6 phage in the CC133 clotting strain 9091) and Sa8 phage in the CC151clotting strain IL83 (fig. S9) which were both confirmed by Nanopore long-read sequencing (table S4). We identified three genes that were present in the clotter and absent in the nonclotter of both pairs 1 and 3, but the same pattern was observed in the pair 3 control, and overall, the variation in gene complement did not provide an explanation for the loss of clotting phenotype (table S4). For the SNP analysis, a total of 120 SNPs was identified that affect up to 105 genes, including 18 intergenic SNPs within 100 bp of the CDS start codon. There were 11 to 55 SNPs per pair (19 to 71 in controls). For the 102 SNPs that occurred within CDSs, 10 resulted in frameshifts, 88 were missense variations that altered one or more amino acids, 2 resulted in a gained or lost stop codon, and 2 were indels that did not alter the reading frame (Fig. 5D and table S4). There were no SNPs identified that could explain all four LOF events (Fig. 5D). Within the two LOF events of the CC151 lineage, three genes contained different SNPs, the hypothetical protein group_645, *tarM* and *sarZ* (the two missense variants identified in the GWAS). In addition, *gcvPA* contained different SNPs in three and four of the LOF events, respectively, and *agrC* contained unique SNPs in all four pairs. *gcvPA* encodes a glycine dehydrogenase involved in multiple metabolic pathways and contained distinct missense SNPs in three of the four pairs, with no SNPs identified in any of the control pairs. Notably, for *agrC*, distinct frameshift mutations at nucleotide positions 488 and 1155 were identified in both nonclotting strains belonging to CC151 with the CC133 nonclotting strain having a missense variant at nucleotide position 487 and the CC188 nonclotting strain encoding a premature stop codon in *agrC* at nucleotide position 152. Sequence alignment of the *agrC* gene sampled from across the dataset identified an additional three nonclotting strains in CC350 that contained an identical frameshift mutation at nucleotide position 488 or 487 (Fig. 5E). Combined with the *sarZ* GWAS variants identified, these data support the hypothesis that reduced or loss of *agr* expression may result in sporadic loss of milk clotting phenotype in bovine *S. aureus* populations. Accordingly, we tested this further by complementing natural *agr*-deficient bovine *S. aureus* nonclotting strains with expression plasmids encoding the entire *agr* operon. The introduction of a functional *agr* system to nonclotting ST133 and ST151 strains resulted in enhanced Aur expression and conversion to a clotting phenotype (Fig. 6). These data are consistent with a key role for *agr* in control of aureolysin expression as previously reported (*30*).

Overall, our genome-wide and comparative genomic analysis has revealed molecular correlates of sporadic loss of the clotting phenotype. In addition, the data indicate that the emergence of the clotting phenotype during the evolutionary history of bovine *S. aureus* has occurred via distinct genes or gene mutations in different strain backgrounds in parallel.

## DISCUSSION

As a generalist, *S. aureus* has the capacity to adapt to a wide array of host species and has undergone numerous host-switching events during its evolutionary history, particularly between humans and cattle (*21*). In total, 14 major contemporary bovine-associated lineages have been identified that have resulted from at least four human-to-bovine host jumps (*31*). In some cases, host jumps back into humans have led to the emergence and spread of human epidemic clones (*32*). Accordingly, *S. aureus* represents an excellent model for exploring the adaptation of a generalist bacterial species to a new host niche. Here, we have identified that bovine strains of *S. aureus* have undergone adaptive metabolic remodeling compared to human strains of the same lineage (Fig. 2). Our findings support a model whereby bovine *S. aureus* have evolved increased capacity to use the abundant carbohydrate lactose and synthesize BCAAs and arginine. Considering that free BCAAs and arginine are present in milk (*33*), it is unclear why these biosynthetic pathways should be up-regulated in bovine compared to human strains, especially as they are not usually activated during in vitro conditions (*34*, *35*). However, consistent with our findings, previous studies have reported elevated levels of BCAAs in milk during infection by *S. aureus*, and this is now being explored as an alternative marker for subclinical mastitis (*36*). In addition to protein synthesis, an array of important processes has been linked to BCAA biosynthesis, including the production of branched-chain fatty acids and the regulation of virulence by CodY (*37*) or Sae (*38*).

After milk clotting, underpinned by increased expression of Aur by bovine strains, the metabolic remodeling of bovine *S. aureus* promotes enhanced growth in milk (Figs. 1C and 4E). The discovery of an Aur-dependent growth effect provides an understanding of how Aur contributes to nutrient acquisition in different ecological niches. Aur is already known to be important during abscess infection through the generation of collagen peptides as nutrients for growth, highlighting the broad relevance of Aur for the metabolic adaptation of *S. aureus* in different tissue types and host species (*39*). The importance of peptide metabolism by a human *S. aureus* strain in milk has been reported previously, with a second phase of growth attributed to the release of amino acids by casein digestion in an SspA- and oligopeptide transporter (Opp3)–dependent manner (*40*). In the current study, we demonstrate that the enhanced growth in milk by bovine strains is Aur rather than SspA dependent (Fig. 3D) and likely due to the proteolysis of casein (Fig. 4, A to C), the most abundant protein in milk. The elevated expression of Aur in milk clotting strains (Fig. 3B) is not dependent on genetic alterations to the *aur* gene or its promoter but instead different mutations at distinct gene loci in different strain backgrounds have led convergently to the same phenotype. GWAS was not successful in identifying the genetic basis for the clotting phenotype possibly because of the absence of the same underpinning mutations in different lineages. However, the limited number of genomes that could be included and uneven distribution of the phenotype across the population both limit the power of GWAS to detect associations, especially for rare variants. In contrast, GWAS did reveal LOF mutations associated with *sarZ*, a transcriptional promoter of the accessory gene regulator (agr) in nonclotting strains. Furthermore, a variety of frameshift or nonsense mutations was identified in *agrC* nonclotting strains in the pairwise LOF analysis. Agr is a quorum sensing transcriptional regulator that influences the expression of a wide array of virulence factors including proteases (*41*). The regulation of Aur by *S. aureus* is highly complex with numerous integrated regulatory networks including the regulons of two-component systems, influencing Aur expression (*42*). Complementing these predicted *agr* truncations with an intact *agr* was sufficient to restore the milk clotting phenotype through enhanced Aur expression (Fig. 6). Our data suggest that *agr* is required for the enhanced expression of aureolysin, but distinct mutations affecting different genes or pathways have led to the up-regulation of aureolysin in clotting strains from different genetic backgrounds. Overall, our data support the existence of multiple possible evolutionary trajectories to the same adaptive phenotype in different lineages.

Although the great majority of bovine strains from different lineages had the clotting and growth enhancement phenotype, the dominant established global clones CC97 and CC151 had a higher frequency than the more recently emerged and more locally distributed clones e.g., CC1, CC30, CC45, CC188, and CC350. We speculate that this difference in the frequency of the clotting phenotype in different lineages may reflect incomplete adaptation of these clones to the dairy ecological niche (*16*) or that the enhanced expression of aureolysin may have a fitness cost in nonmilk-associated niches during skin colonization or mammary epithelial intracellular growth, thereby selecting for sporadic LOF. These hypotheses could be tested in future studies.

In conclusion, our data reveal how bovine *S. aureus* has undergone convergent remodeling of existing metabolic pathways to pivot to unlock nutrients from substrate specific to the bovine host milieu. These findings highlight the evolutionary plasticity of *S. aureus* that promotes its remarkable capacity to expand into new host niches.

## MATERIALS AND METHODS

### Bacterial strains and growth conditions

All isolates used for genetic and phenotypic analysis are listed in table S1. Mutant strains generated in this study are described in table S5. *S. aureus* strains were routinely cultured in TSB at 37°C with shaking at 180 rpm or statically on tryptone soya agar. Supplementation with chloramphenicol (10 μg/ml) was used as appropriate. For cloning, *E. coli* strains were cultured in LB supplemented with ampicillin (100 μg/ml) or chloramphenicol (15 μg/ml).

### Milk clotting phenotype and assessment of pH

Five milliliters of Arla Cravendale filtered whole milk was inoculated with 1:100 TSB overnight culture, concentrated supernatant, or recombinant proteins and incubated for up to 24 hours at 37°C; 180 rpm. For 96-well plate analysis, overnight cultures were standardized to OD_600_ (optical density at 600 nm) of 0.05 in 200 μl of Arla Cravendale filtered whole milk. The pH was measured by centrifuging the sample and using a pH meter to measure the pH of the whey or unseparated milk. A clotting phenotype was assigned if separation of the milk into whey and clot was observed or if a solid milk clot had been produced upon tilting the tube. Each strain was tested in three different milk sources.

### Growth analysis in milk

Overnight TSB cultures were standardized to a starting OD_600_ of 0.05 in 500 μl of Arla Cravendale filtered whole milk and incubated at 37°C with shaking at 180 rpm for the noted time. For some experiments, 500 μl of TSB was inoculated in the same manner as a comparison. At each time point, the presence of milk clots were noted before addition of 0.02% (w/v) SDS and 10-min vortex at max speed to separate any clots before colony-forming unit (CFU) analysis. Each strain was tested in three different milk batches.

### Data collection

For genomic analysis, we included 358 whole-genome sequence (WGS) datasets that were previously uploaded to public repositories under the codes listed in table S1. Seven additional WGS datasets were uploaded to National Center for Biotechnology Information under the new project code PRJNA1153292. For 347 sets of reads, we used the draft genome assemblies that were generated previously (*17*, *21*, *43*), which were assembled from reads following the protocols described in their respective projects as indicated in table S1. The remaining 18 read sets were trimmed using Trimmomatic (v0.36) and assembled using SPAdes (v3.13.0). Before variant calling, we checked the assembly quality of all collected genomes with QUAST (v4.6.3) (*44*) using the RF122 reference genome (AJ938182) to measure assembly statistics. To avoid artifacts due to highly fragmented assemblies, genomes were excluded from the GWAS and comparative analysis if they had an N50 value below 200,000, more than 50 contigs, or they aligned with less than 80% of the reference genome (table S1).

### Phylogenetic analysis

We used snippy (v4.4.5, https://github.com/tseemann/snippy) to identify SNPs with a reference genome for RF122 (AJ938182), generating pseudo-reads from the draft assemblies with the --ctgs parameter. Using snippy-core to generate an alignment, we filtered out recombined regions from the alignment using gubbins (v2.3.4) (*45*) and extracted core SNPs using snp-sites (v2.5.1) (*46*). The core SNPs were used to generate a maximum-likelihood phylogeny with IQtree (v2.0.5) (*47*), using a general time reversible model of evolution with a four-site gamma distribution to model differences in the evolutionary rate between branches. The tree was generated using the ultrafast bootstrap method with 5000 replicates and optimized on the bootstrap alignment using nearest-neighbor interchange with the --bnni parameter. Ascertainment bias correction was applied to prevent the overestimation of branch lengths by excluding invariant sites, and polytomies were collapsed with the --polytomy parameter.

### RNA sequencing experimental setup and analysis

To facilitate RNA extraction, 50% Arla Cravendale filtered whole milk was used diluted in TSB. Overnight cultures were standardized to OD_600_ of 0.1 in TSB cultures and OD_600_ of 0.25 in 50% milk/TSB cultures in 5-ml volumes, incubated at 37°C at 180 rpm for 2 hours before pelleting at 4863*g* for 5 min. The pellet was resuspended in 200-μl Tris-EDTA (TE) buffer and transferred into a 96-well plate before centrifugation. The supernatant was removed, and the pellets were immediately frozen on dry ice containing 100% ethanol. The frozen pellets were briefly stored at −80°C before shipment to Genewiz, UK for RNA extraction and paired-end RNA sequencing.

Raw data reads have been deposited in ArrayExpress (E-MTAB-14384). Bowtie2 v2.4.2 (*48*) was used to map sequence reads to a corresponding indexed reference genome built using bowtie2-build. Before reads were mapped, Cutadapt v1.16 (*49*) trimmed adapters. Samtools v1.10 (*50*) was first used to convert the sequence alignment and map files, outputted by Biowtie2, to a binary alignment and map (BAM) file and then to sort BAM files by genome position. CoverageBED (BEDtools v2.30.0) (*51*) generated count data files from sorted BAM files. Analysis of differential gene expression between groups of different strains required shared genes across all genomes to be identified and count data files adjusted so that all shared genes had the same name across all count data files. To identify shared genes across all genomes, Roary v3.6.2 (*52*) was used to calculate a pan genome. The core genes were defined as genes with >95% sequence identity. For duplicated genes, Roary assigned multiple genes within a genome to a single core gene, and the first gene listed by Roary was arbitrarily taken as the gene representing this core gene. Hence, only the count data for this gene, and not the other duplicates, were included in later analysis. In the later analysis of differential expression data, none of the genes of interest represented duplicated genes. The DESeq2 (Bioconductor v3.13) (*51*) package was used to generate differential expression data.

### Aureolysin Western blot analysis

Aur-specific IgY antibody was generated from immunization of two hens with two 16–amino acid peptides (CYYKDTFGRESYDNQG and CEGDALRSMSNPEQFG) of Aur by Eurogentec. Antibody was affinity-purified from eggs after three antigen boosts. For Western blot analysis, *S. aureus* was cultured for 5 hours in TSB before centrifugation and concentration of supernatant using Amicon Ultra Centrifugal units (10-kDa cutoff). Supernatant protein content was determined using the Pierce Coomassie Plus (Bradford) protein assay kit before separation by SDS–polyacrylamide gel electrophoresis (SDS-PAGE; 4 to 20% Mini-PROTEAN TGX Pre-cast Protein gels, Bio-Rad). Blotting was performed using a Trans-Blot Turbo RTA Mini 0.2 μm Nitrocellulose transfer kit (Bio-Rad). After the transfer, Revert 700 Total Protein Stain reagent (LI-COR) was used according to the manufacturer’s instructions to stain and acquire the total protein content per sample on the membrane using the Licor Odyssey M. Blots were then blocked in 8% (w/v) semiskimmed milk powder in phosphate-buffered saline (PBS) for at least 2 hours at room temperature (Sigma-Aldrich) before adding 15 μg of anti-Aur IgY antibody overnight at 4°C. After washing, 2 μg of F(ab) goat anti-chicken immunoglobulin G horseradish peroxidase (Sigma-Aldrich) was applied for 1 hour in 0.05% PBS–Tween 20. Reactive bands were visualized using ECL and the Syngene GeneGnome. Quantification of Aur signal was performed in Fiji (ImageJ) and normalized against the total protein content of the respective sample. Normalization analysis was performed on at least three biological replicates.

### Generation of protease expression, *agr* complementation, and deletion mutant constructs

For protease expression constructs, *aur* or *sspA* genes were amplified with their native promoter and ribosomal binding site (table S6) using Q5 polymerase [New England Biolabs (NEB)]. pCT was generated by integration of 6xHis-tag and Strep-tagII into pALC2073 (*25*). pCT was digested with SacI-HF and EcoRI-HF and Antarctic Phosphatase treated (NEB), before gene cloning using Gibson assembly (NEB), and introduced into *E. coli* IM08B (*53*) and subsequently into relevant *S. aureus* strains (table S5). Constructs were confirmed by Sanger sequencing (Eurofins).

For *agr* complementation, the whole *agr* operon encoding RNAIII was amplified with the native promoter and ribosomal binding site (table S6) using Q5 polymerase (NEB). Constructs were generated in the expression plasmid pALC2073 (*25*), digested with BamHI-HF and SacI-HF (NEB) and Antarctic Phosphatase treated (NEB), using T4 DNA ligase (NEB) and introduced into *E. coli* DC10B (*54*) and subsequently into relevant *S. aureus* strains (table S5). Constructs were confirmed by whole plasmid sequencing (MicrobesNG).

Allele replacement was performed using thermosensitive pIMAY-Z (*53*) using protocols previously described (*54*). Briefly, oligonucleotide primers were used to amplify ~500-bp flanking regions of *aur* or *sspA* genes (table S6) using Q5 polymerase (NEB) and *S. aureus* genomic DNA (Monarch genomic DNA purification kit, NEB). Gibson assembly (NEB) and transformation into *E. coli* DC10B (*54*) allowed insertion of AB and CD polymerase chain reaction products into pIMAY-Z digested with KpnI and NotI/SacI. After confirmation of construct generation by Sanger sequencing (Eurofins), plasmids were transformed into relevant *S. aureus* strains (table S5). After temperature shifting, gene deletions and the absence of spurious mutations that could affect phenotype were confirmed through whole-genome sequencing (MicrobesNG).

### Purification of Aur from *S. aureus* supernatant

Aur was purified from the concentrated supernatant of USA300Δprotease pCT::*aur* dialyzed in binding buffer [10 mM tris (pH 7.5), 5 mM potassium chloride, 5 mM calcium chloride, and 1.5 mM zinc chloride (pH 8)]. Zinc had to be maintained throughout the purification process to guarantee functional purified Aur. Anionic purification was performed using a HiTrap DEAE FF column (Cytiva) with two wash buffers [wash buffer 1: 10 mM tris (pH 7.5), 50 mM potassium chloride, 2.5 mM calcium chloride, and 1.5 mM zinc chloride (pH 8); wash buffer 2: 10 mM tris (pH 7.5), 200 mM potassium chloride, 2.5 mM calcium chloride, and 1.5 mM zinc chloride (pH 8)] and a final elution buffer [10 mM tris (pH 7.5), 600 mM potassium chloride, 2.5 mM calcium chloride, and 1.5 mM zinc chloride (pH 8)]. Positive fractions were dialyzed to 10 mM tris (pH 7.5), 5 mM potassium chloride, 2.5 mM calcium chloride, 1 mM zinc chloride, and 100 mM sodium chloride (pH 8) using a 3.5 molecular weight cut-off (MWCO) G3 dialysis cassette (Thermo Fisher Scientific) at 4°C. Recombinant SspA protein was purchased from Sigma-Aldrich (P6181).

### Digestion of casein

To generate concentrated supernatant samples, USA300Δprotease pCT::*aur* and KVSA-7 pCT::*sspA* (this ST97 bovine nonclotting strain was used because USA300Δprotease pCT::*sspA* does not express functional SspA) were induced at mid-exponential phase with anhydrotetracycline (3 μg/ml) and cultured overnight. The supernatant was concentrated and dialyzed to PBS using Amicon Ultra centrifugal units (10-kDa cutoff) (fig. S7). Protein concentration was quantified using the DS-7 UV-Vis Spectrophotometer (DeNovix). The proteolytic activity of 1 μM concentrated supernatant or recombinant protein was compared using FTC-casein (Pierce Fluorescent Protease Assay Kit, Thermo Fisher Scientific). The manufacturer’s instructions for a microplate assay were followed with a 30-min reaction time before reading on the CLARIOstar plate reader (BMG LABTECH).

To assess the ability to digest all three casein chains, 2 μg of each α-casein, β-casein, and κ-casein chains of bovine origin (Sigma-Aldrich) were incubated with 4 μg of protein for 1 hour 30 min at room temperature and then analyzed by SDS-PAGE (4 to 20% Mini-PROTEAN TGX Pre-cast Protein gels, Bio-Rad) for cleavage. A total of 25 mM EDTA was used as a control as it inhibits the activity of Aur but not SspA.

### Genome-wide association analysis

To perform the GWAS, unitig-caller (v1.3.1) (https://github.com/bacpop/unitig-caller) was used to call unitig features across 254 high-quality genome assemblies, using 31 bp as the k-mer length to construct the de bruijn graph. GWAS was then conducted using a linear mixed-effects model in pyseer (v1.3.6) (*55*), with the host and phylogenetic distances included as covariates in the model. To control for the population structure, a pairwise-kinship matrix was generated from the phylogeny using the phylogeny_distance.py pyseer script and provided as covariates in the model using the --similarity parameter. Following the GWAS, unitig-caller query mode was used to map the distribution of significant unitigs across the population. For the Manhattan plots, all unitigs were mapped to the closest gene in a reference genome using the annotate_hits_pyseer script packaged with pyseer. In the order in which they were used to map variants, we included NCTC8325 (GCA_000013425.1), RF122 (GCA_000009005.1), and ED133 (GCA_000210315.1) as reference genomes. For unitigs that were absent from these reference genomes, the GWAS input data were annotated using PROKKA (v1.14.6) (*56*) and searched in alphanumeric order using the “draft” option to allow for close, nonspecific matching of the remaining unitigs. PROKKA was run with the --compliant and --force parameters, the --centre parameter listed as “UoE” and the --genus specified as “Staphylococcus.” A second GWAS was conducted by the same method on just the 104 CC97 genomes in the dataset.

### Comparative genomic analysis of gain or loss of clotting events

To identify the full complement of genomic differences between pairs of isolates, we combined variant calling with accessory genome analysis. Variant calling was performed with snippy (v4.4.5), using the PROKKA .gbk file for the nonclotting strain as the reference and the contigs for the clotting strain as the query with the --ctgs parameter. For each of the six pairs analyzed, the same exact analysis was repeated for a closely related control pair with a similar number of SNPs. The closest gene was identified for each SNP using the closest script in bedtools (v2.29.2) (*57*), converting gene gff files to .bed files using AGAT (v0.8.0, https://github.com/NBISweden/AGAT) (*58*). All gene sequences were extracted from PROKKA output using an in-house gff parsing script (https://github.com/JPegorino/GF-effortless) (v0.0.0) and translated to protein sequence using the transeq script in EMBOSS (v6.6.0) (*59*). To investigate the broader patterns of mutations in *agrC* and *sarZ* across the GWAS dataset, we inspected CDS nucleotide alignments generated by mafft (v7.525) with the --auto flag.

To avoid potential sequence assembly artifacts, we filtered out variants from contigs of <5 kb in length from downstream analysis and those that mapped to the repeat region of cell wall–associated proteins that have highly polymorphic stretches of serine-aspartate residues (*60*). Last, to focus on variants most likely to explain the phenotype, we filtered the data to include only nonsynonymous variants located up to 100 bp upstream of a gene sequence and removed variants that were found in both experiment and control data for a pair. CDS nucleotide sequences corresponding to the variants present after filtering are provided in file S1.

To identify larger indels and accessory regions, we used Mauve (snapshot 2015-02013) (*61*) to reorder the contigs in both isolates to best match the sequence in a closely related reference, selected from a database of complete *S. aureus* genomes in Refseq by minimum mash distance [calculated with mash v2.3 (*62*)]. Links to each selected reference in Refseq are included in table S4. Aligned contigs were then combined into single sequences and compared manually using the Artemis Comparison Tool (Release 18.1.0) (*63*), creating .gff files that marked on the contig breaks by adapting the code in https://github.com/widdowquinn/scripts/blob/master/bioinformatics/stitch_six_frame_stops.py. To identify accessory genes, panaroo (*64*) was used for each genome pair, and the output was matched up the data to CDSs in the combined assembly FASTAs by reannotating with PROKKA (*56*), keeping the same parameters except for providing the original annotations as trusted annotations for the --proteins parameter. To confirm the presence and locations of prophage and transposable element sequences that were predicted to differ by our analysis in Artemis comparison tool (ACT), we performed Nanopore long-read sequencing of the relevant pairs of isolates with opposing phenotypes. Sequencing and assembly of these genomes were performed externally by MicrobesNG. Long-read assemblies were annotated using PROKKA (v1.14.6) with --centre x and --compliant parameters. Panaroo (v1.5.2) was then run for each pair to confirm accessory genes, setting the --family_threshold to 0.95 and the --core_threshold to 100 but otherwise keeping default parameters. Prophage sequences were also confirmed using PHASTER (https://phaster.ca/) (*65*) Long-read sequences were uploaded to European nucleotide archive (ENA) and have been made publicly available under project code PRJNA1153292.

To complement our analysis of pairs that had gained the clotting phenotype, we selected emergent clades (with >85% UFBoot support) from the phylogeny that had gained the phenotype within a broader lineage (>95% UFBoot support) that lacked it (fig. S8). We limited this analysis to two clades in which all isolates shared the clotting phenotype and for which there existed a sister clade (with >95% UFBoot support) of isolates lacking the phenotype that could be used as a control. To analyze these clades alongside the pairs, we called SNPs for each isolate in each clade against an outgroup (100% UFBoot support), selected by the highest N50 out of the set of genomes that were close outgroups to the clade. We then filtered one of the Snippy output tables for a random isolate to include only variants shared with all the others in the clade. This table was then matched with genes and filtered in the same way as the variant tables for the pairs, including the removal of any identical variants shared with the control data. Because of the inability for ACT to compare more than two genomes, we could not analyze the accessory genome elements in these clades by the same method as for pairs. To determine the shared accessory gene content between pairs/clades and with respective controls, panaroo (v1.5.0) was run with the same parameters used to analyze the individual long-read pairs. For this analysis, we included the long-read sequence annotations for pairs with opposing phenotypes and the GWAS annotations for corresponding control pairs. For the two clades, all isolates from both test and control clades were included, and the data were filtered to exclude presence/absence patterns that were not conserved throughout the clade before comparing with the data for pairs.

### Statistical analysis

For statistics and data visualization of the RNA sequencing and GWAS data, the programming language R was used in RStudio. Specifically, for the GWAS, a 0.05 significance threshold was selected with a Bonferroni correction for multiple testing applied. We used the pyseer count_patterns script to help calculate Bonferroni correction thresholds and the qq_plot pyseer script to help inform appropriate thresholds for the minor allele frequency (MAF). For the GWAS with 254 genomes, we filtered out hits with MAF < 0.03. For the GWAS with only CC97 genomes, a higher minimum MAF cutoff of 0.1 was used. Manhattan plots were generated using ggplot2 (v3.4.4) in RStudio (v4.4.2). All additional statistical analysis was performed in GraphPad v10. Each dataset was analyzed for normality and data variance before selecting the most appropriate statistical test, which is detailed for each dataset within the figure legends. The following conventions were used for statistical significance: not significant, *P* > 0.05; *P ≤ 0.05; **P ≤ 0.01.
