# Supplementary material for: Bacterial metabolic remodeling by convergent evolution unlocks nutrient availability after a host switch
Source: Sci Adv. 2026 Feb 6;12(6):eadw9419. doi: 10.1126/sciadv.adw9419 (PMC12880543; doi:10.1126/sciadv.adw9419)
Supplement: Supplementary file 1 — Figs. S1 to S9 Legends for tables S1 to S4 Tables S5 and S6 Legend for file S1 [file sciadv.adw9419_sm.pdf]

Science Advances
AAAS

Supplementary Materials for

Bacterial metabolic remodeling by convergent evolution unlocks nutrient availability after a host switch

Amy C. Pickering *et al.*

Corresponding author: J. Ross Fitzgerald, ross.fitzgerald@ed.ac.uk

Sci. Adv. **12**, eadw9419 (2026)
DOI: 10.1126/sciadv.adw9419

The PDF file includes:

Figs. S1 to S9
Legends for tables S1 to S4
Tables S5 and S6
Legend for file S1

Other Supplementary Material for this manuscript includes the following:

Tables S1 to S4
File S1

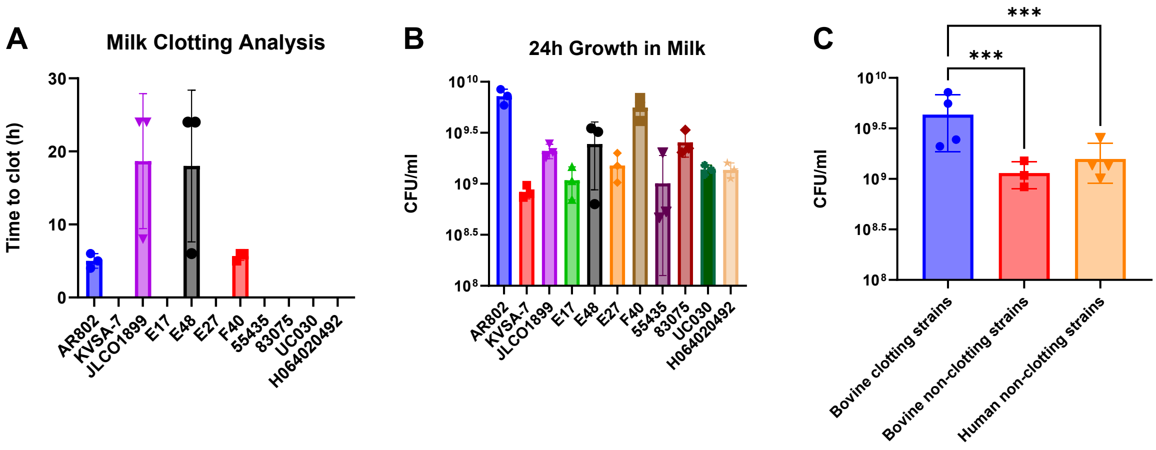

Fig. S1. Milk Clotting and Growth of ST97 strains selected for transcriptomic analysis. (A), Time to clot milk of each strain. If no data points are present then the strain did not clot milk after 24 h of growth at 37°C with shaking. **(B),** CFU analysis of strains cultured in 5 ml of Arla Cravendale filtered whole milk for 24 h at 37°C with shaking. Each data point represents a biological replicate, *n*=3. **(C),** Data is combined for each milk clotting phenotype. Each data points represents *n*=3 biological replicates for each strain. Error bars, means ± standard deviation. Two-way ANOVA, *** $p<0.005$.

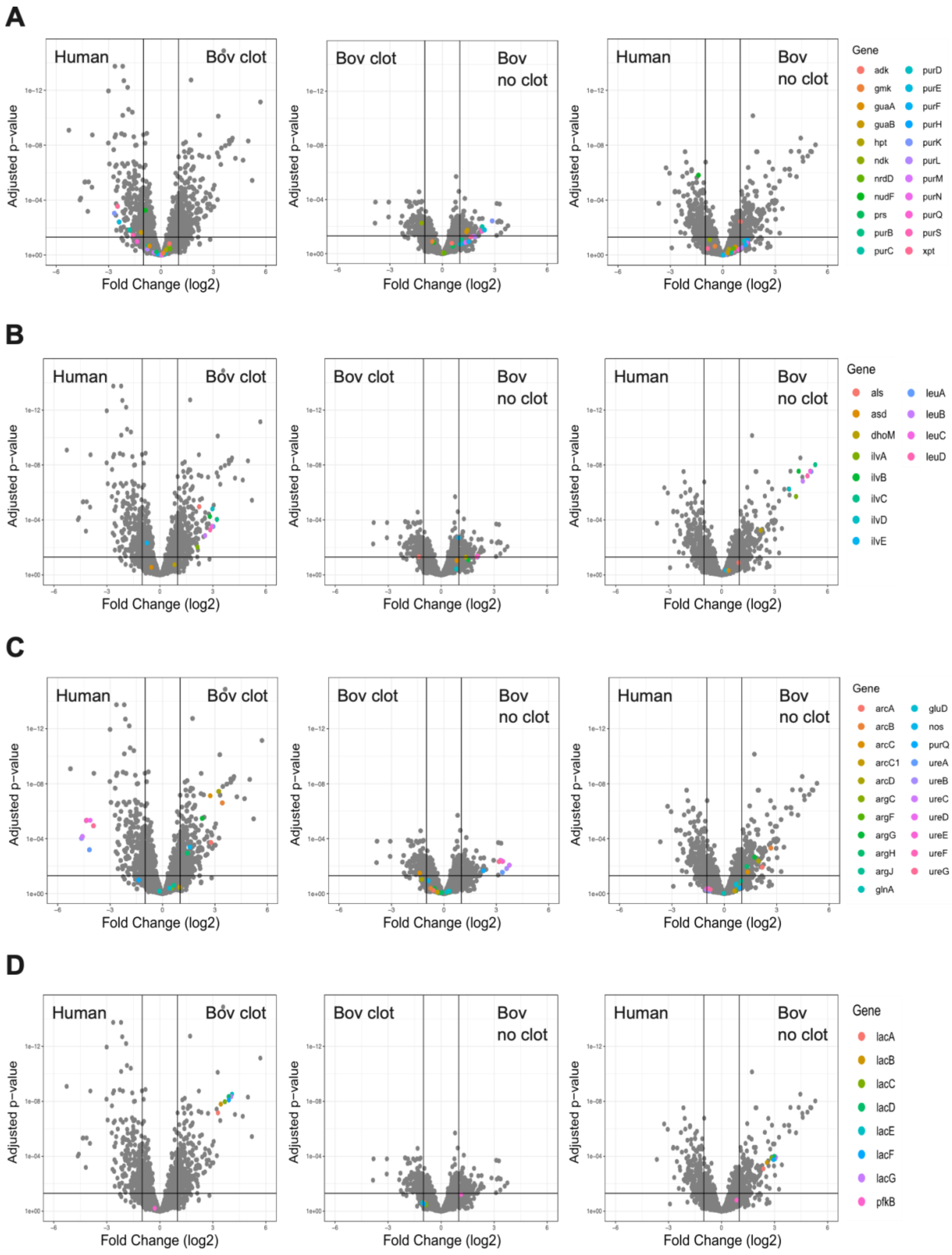

Fig. S2. Volcano plots demonstrating the metabolic pathways differentially expressed between groups in milk. (A), Purine metabolism pathway. **(B),** Valine, leucine, and isoleucine biosynthesis. **(C),** Arginine biosynthesis. **(D),** Galactose metabolism.

A

Purine Metabolism Pathway in RF122

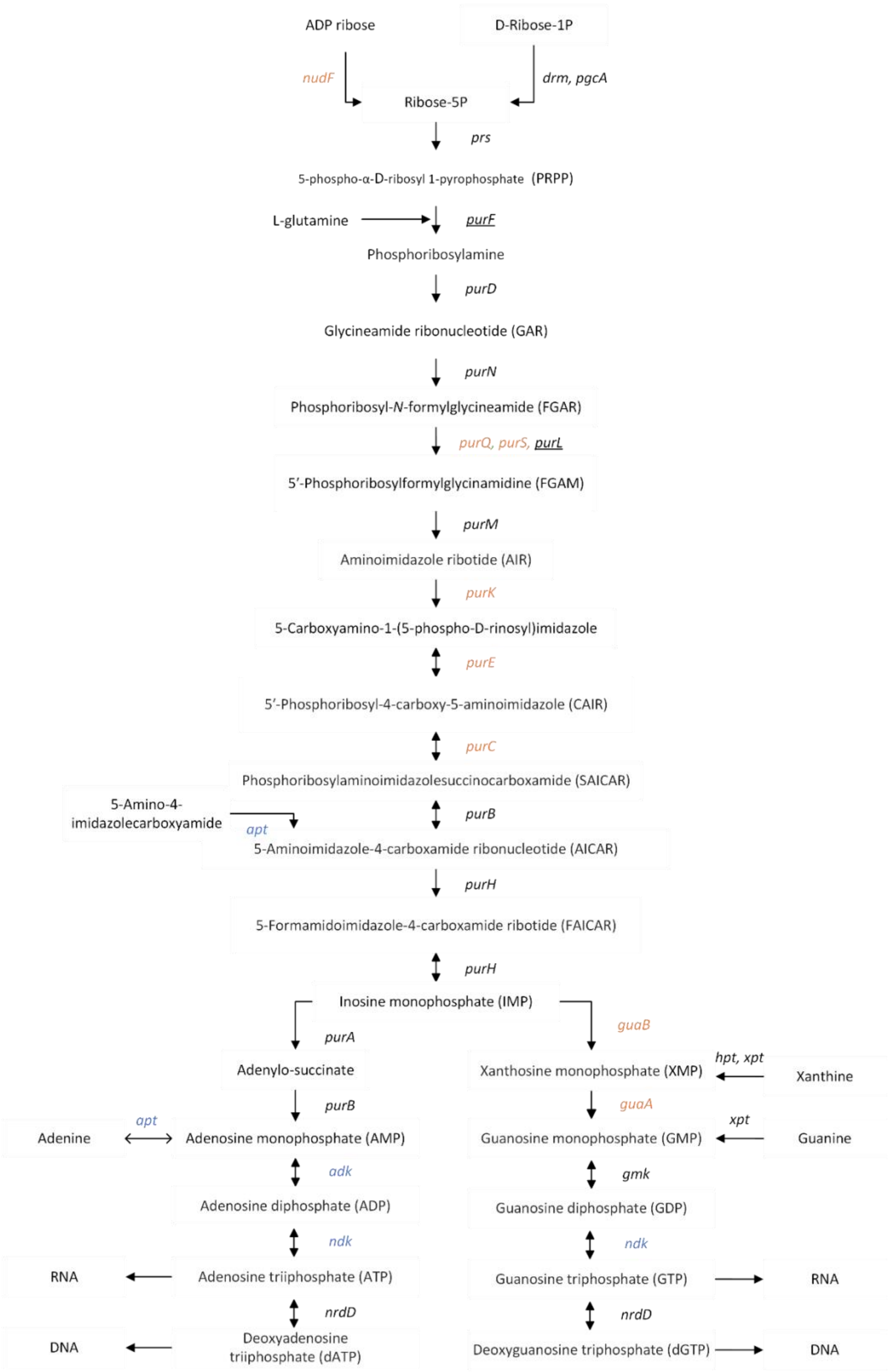

B **Valine, Leucine, and Isoleucine Biosynthesis Pathway in RF122**

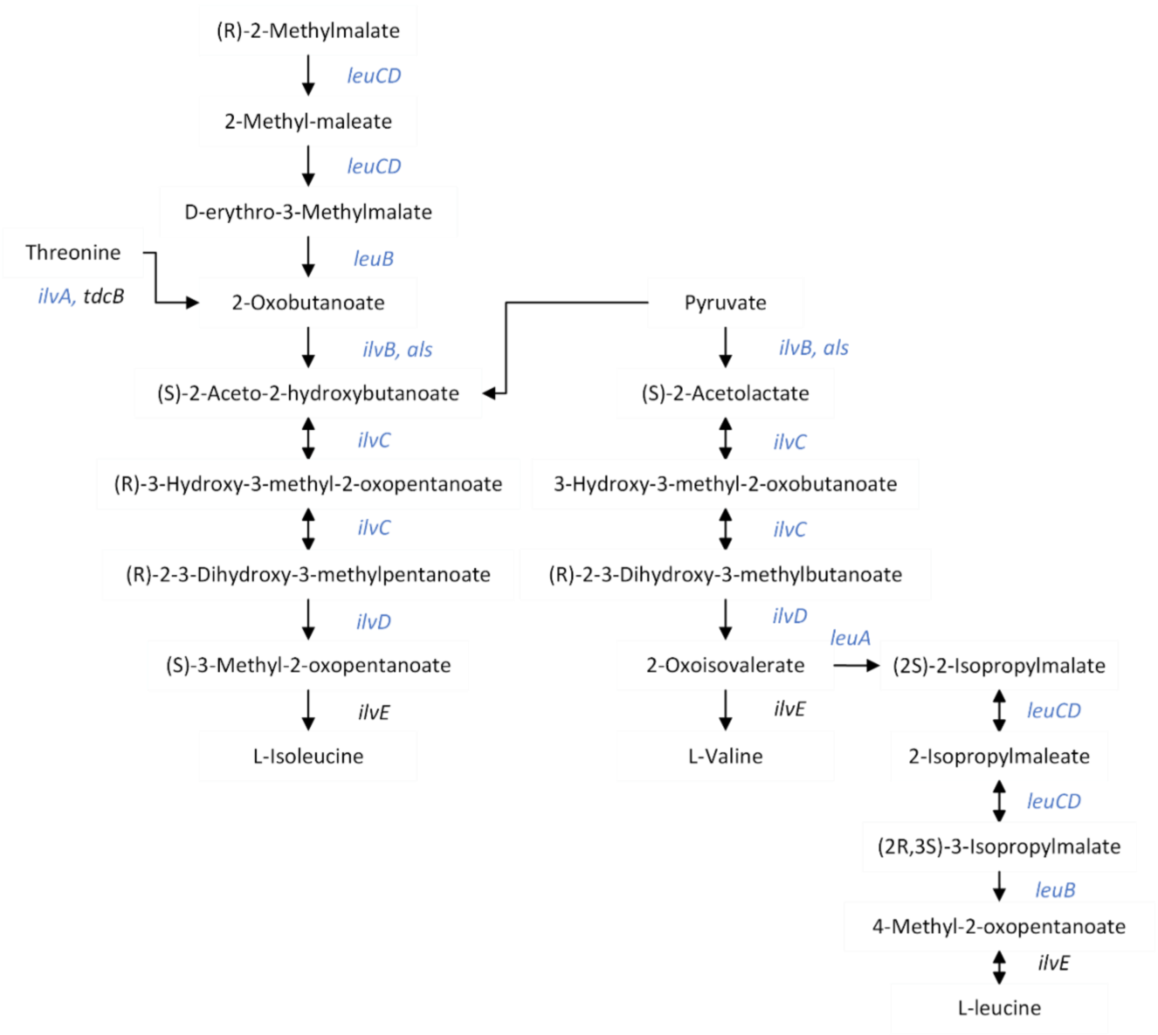

C **Arginine Biosynthesis Pathway in RF122**

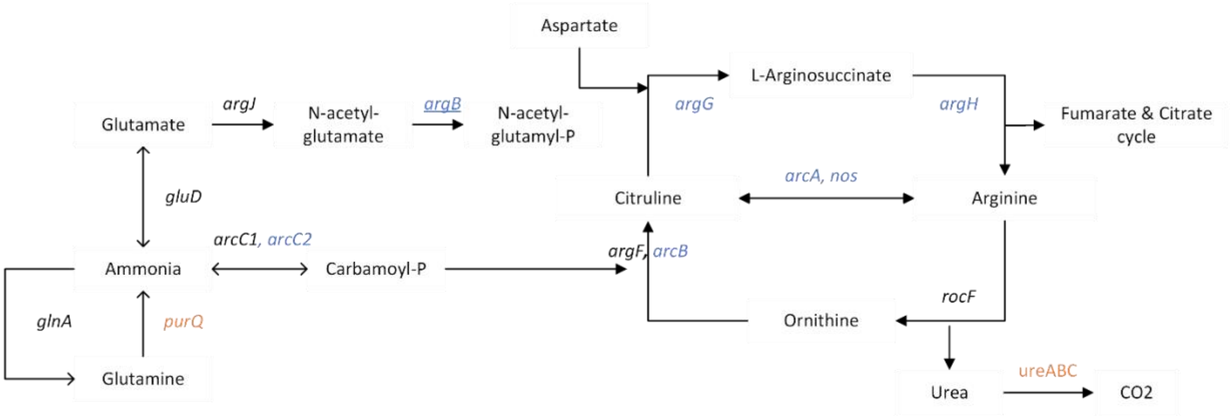

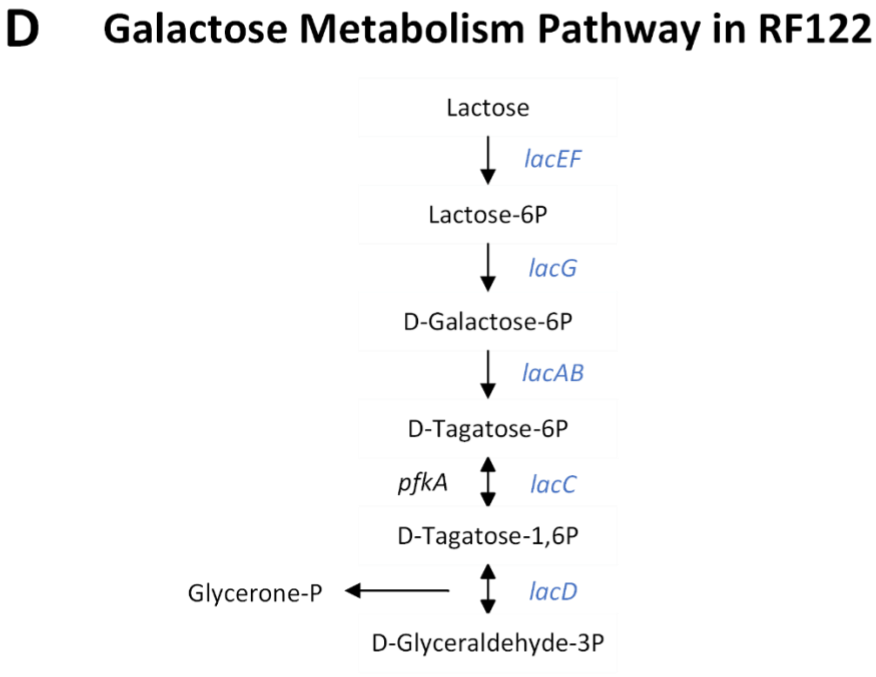

Fig. S3. Schematics of the major metabolic pathways differentially expressed between groups in milk. (A), Purine metabolism pathway. **(B),** Valine, leucine, and isoleucine biosynthesis. **(C),** Arginine biosynthesis. **(D),** Galactose metabolism. Genes colored in orange are more highly expressed in human or non-clotting strains. Genes colored in blue are more highly expressed in bovine strains compared to human strains. Genes that are underlined, were also identified to contain SNPs in the pairwise SNP analysis.

Strain	WT	pALC2073	pCT::*aur*
USA300	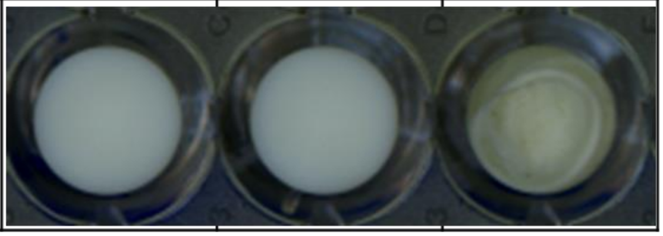		

Fig. S4. Overexpression of aureolysin leads to milk clotting of *S. aureus* USA300. Arla Cravendale filtered whole milk was inoculated with each strain in triplicate and incubated statically at 37°C for 24 h. Milk clotting was assessed visually and imaged using an Epson scanner.

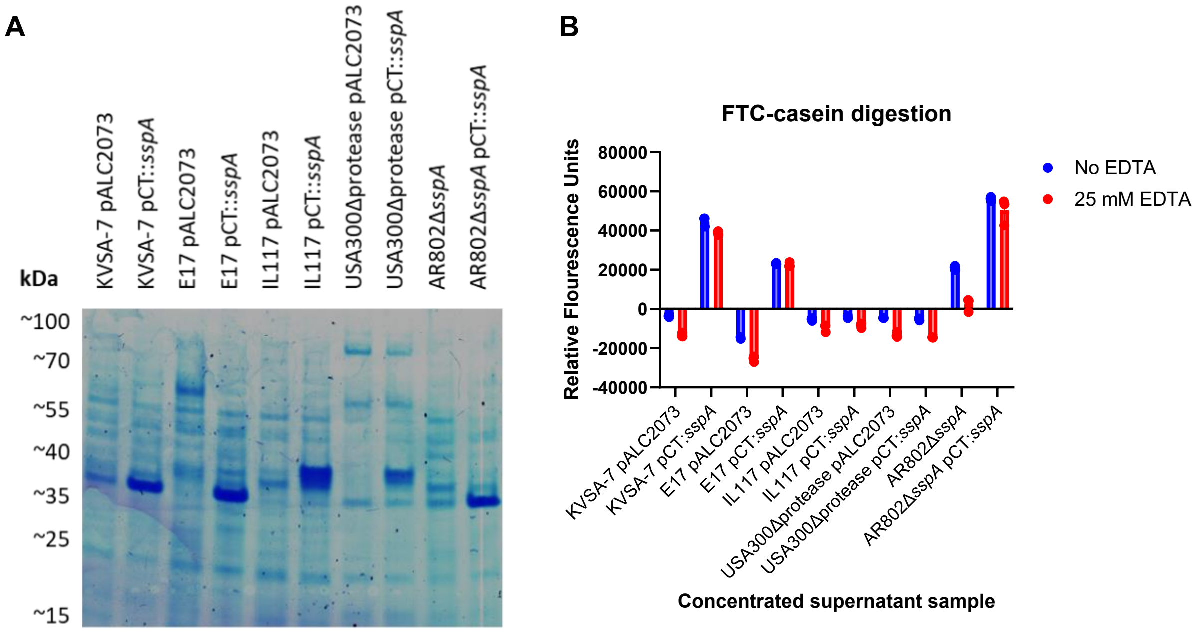

Fig. S5. Overexpression of SspA and casein digestion. (A), Concentrated supernatant samples of strains containing an empty expression plasmid or the SspA expression construct analyzed by SDS-PAGE (4-20% Mini-PROTEAN® TGX™ Pre-cast Protein gel, Bio-Rad). SspA is observed at two molecular weights of ~40 kDa and less than 40 kDa. **(B)**, FTC-casein digestion by the same concentrated supernatant samples. The lower molecular weight SspA protein observed in KVSA-7, E17, and AR802ΔsspA is associated with SspA activated by Aur that is capable of digesting casein in the presence of 25 mM EDTA, which inhibits the activity of aureolysin.

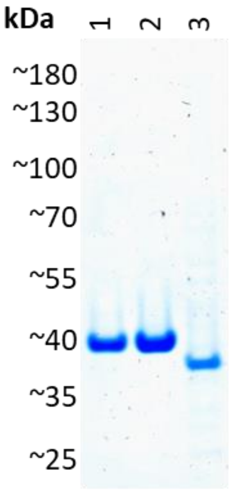

Fig. S6. Concentrated supernatant and purified Aur samples. SDS-PAGE (4-20% Mini-PROTEAN® TGX™ Pre-cast Protein gel, Bio-Rad) showing the protein profile of (1) concentrated supernatant of USA300Δprotease pCT::*aur,* (2) purified Aur, and (3) concentrated supernatant of KVSA-7 pCT::*sspA*.

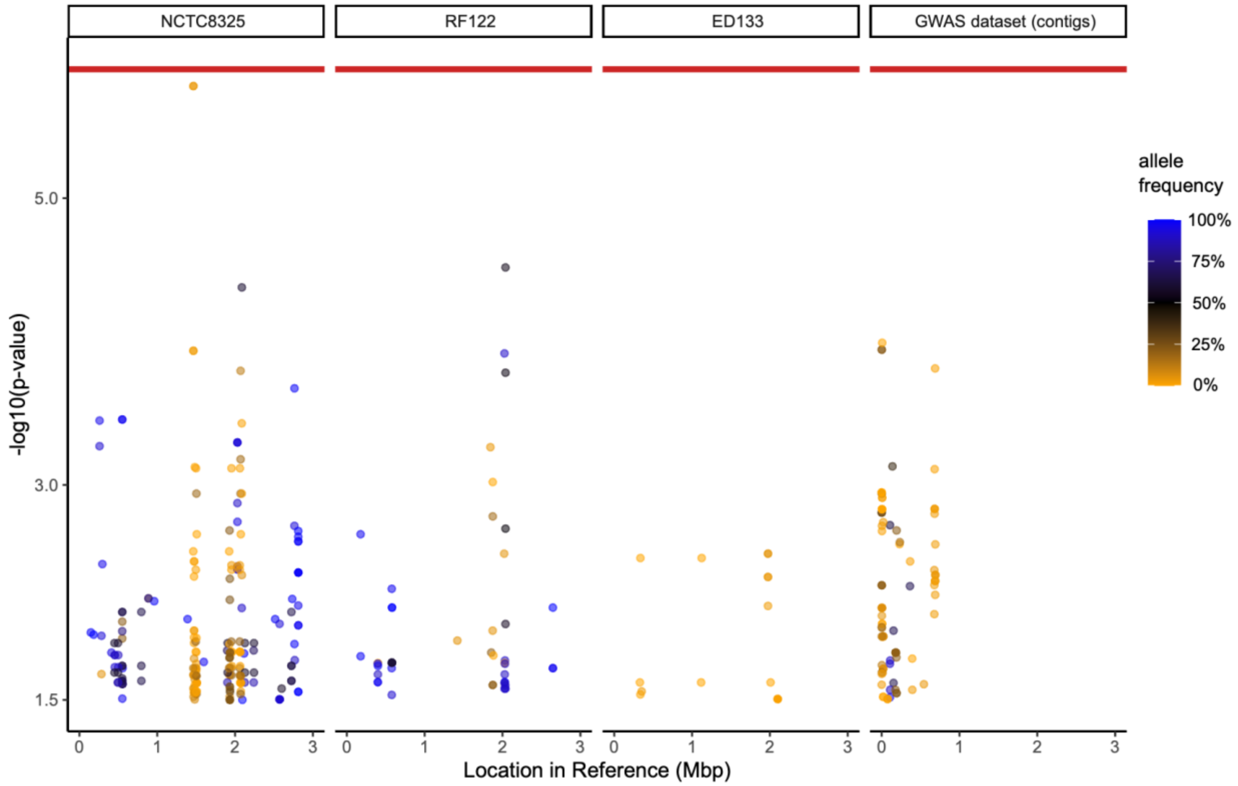

Fig. S7. Genome wide association analysis of the milk clotting phenotype in CC97. A), Manhattan plot showing the significance of association with the milk clotting phenotype across the set of unitigs that constitute 104 genome sequences of CC97 isolates tested for clotting. Unitigs with p-values $>10^{-1.5}$ are not shown. The X axis shows the co-ordinates of each unitig motif in a reference genome containing that motif (Mbp), mapped iteratively as described in the Methods. The Y axis shows the absolute magnitude of p-values for each associated variant. Each point represents a single unitig and is colored proportionally to its frequency across the data, as indicated by the scale bar to the right of the panel. A horizontal red line shows the Bonferroni-corrected significance threshold used (alpha=0.05).

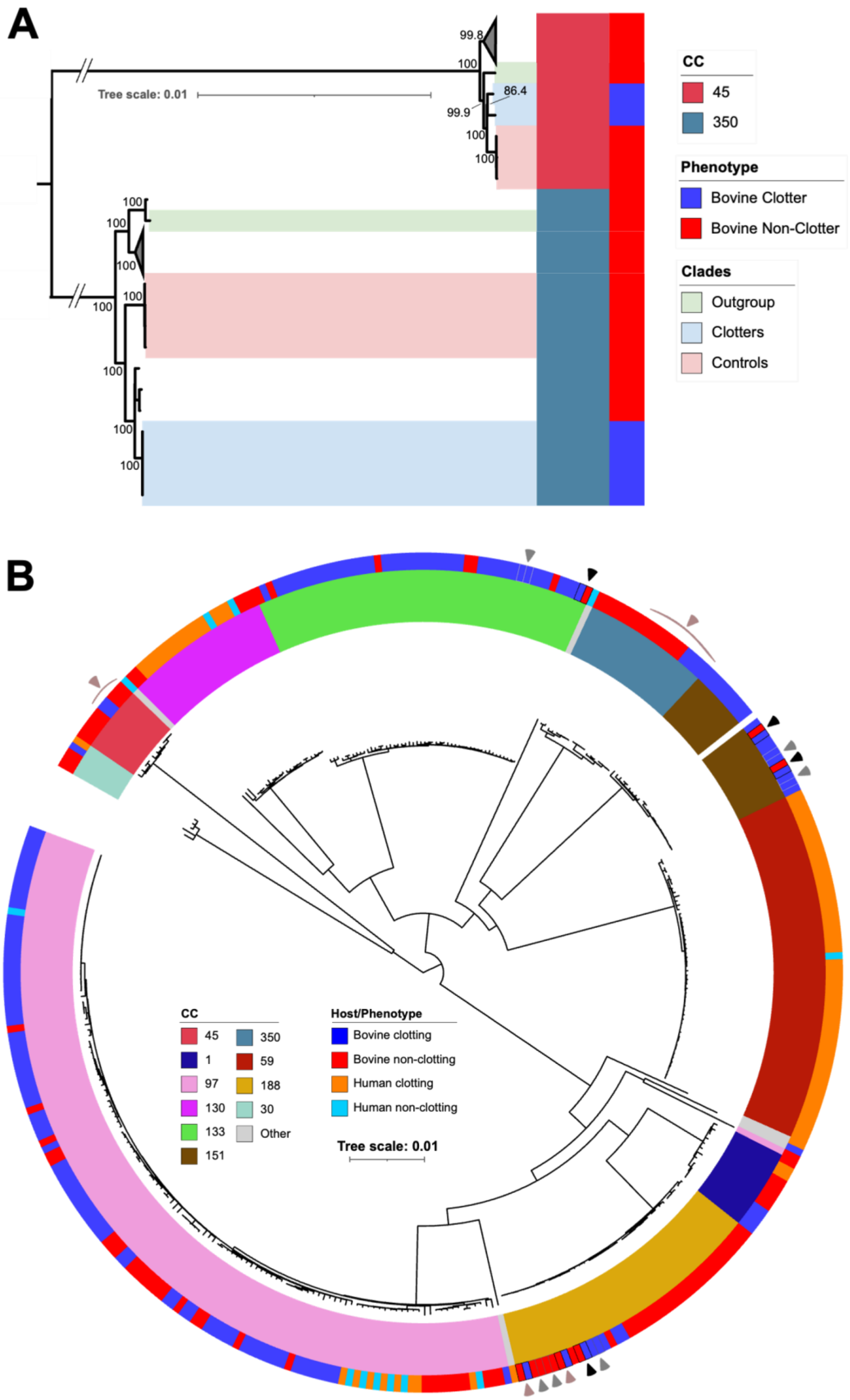
A
Tree scale: 0.01
99.8
100
86.4
99.9
100
100
100
100
100
100
100
100
100
100
CC
45
350
Phenotype
Bovine Clotter
Bovine Non-Clotter
Clades
Outgroup
Clotters
Controls
B
CC
45
1
97
130
133
151
350
59
188
30
Other
Host/Phenotype
Bovine clotting
Bovine non-clotting
Human clotting
Human non-clotting
Tree scale: 0.01

Fig. S8 Phylogenetic topology of gain of function clades and the location of paired analysis isolates in the population. (A), a pruned version of the core SNP phylogeny in Figure 1E (main manuscript) and **B**, showing just the lineages (CCs) encompassing the clades used in the gain of function analysis. Coloured blocks are used to indicate the Phenotype, CC and sub-clade with respect to the analysis according to the figure legends on the right. Clades containing only isolates with the clotting phenotype are labelled as 'Clotters', whilst sister clades lacking the phenotype are labelled as 'Controls'. UFBoot bootstrap support values are indicated at relevant nodes of the tree. Clades that contain no isolates used in the analysis are collapsed. Double dashes indicate that branches have been shortened for legibility. **(B),** the locations of gain of function pairs and clades (tan arrows), loss of function pairs (black arrows) and respective controls for both of these (grey arrows) indicated on the phylogeny from Figure 1E. Matching experiment and control pairs always occur adjacently.

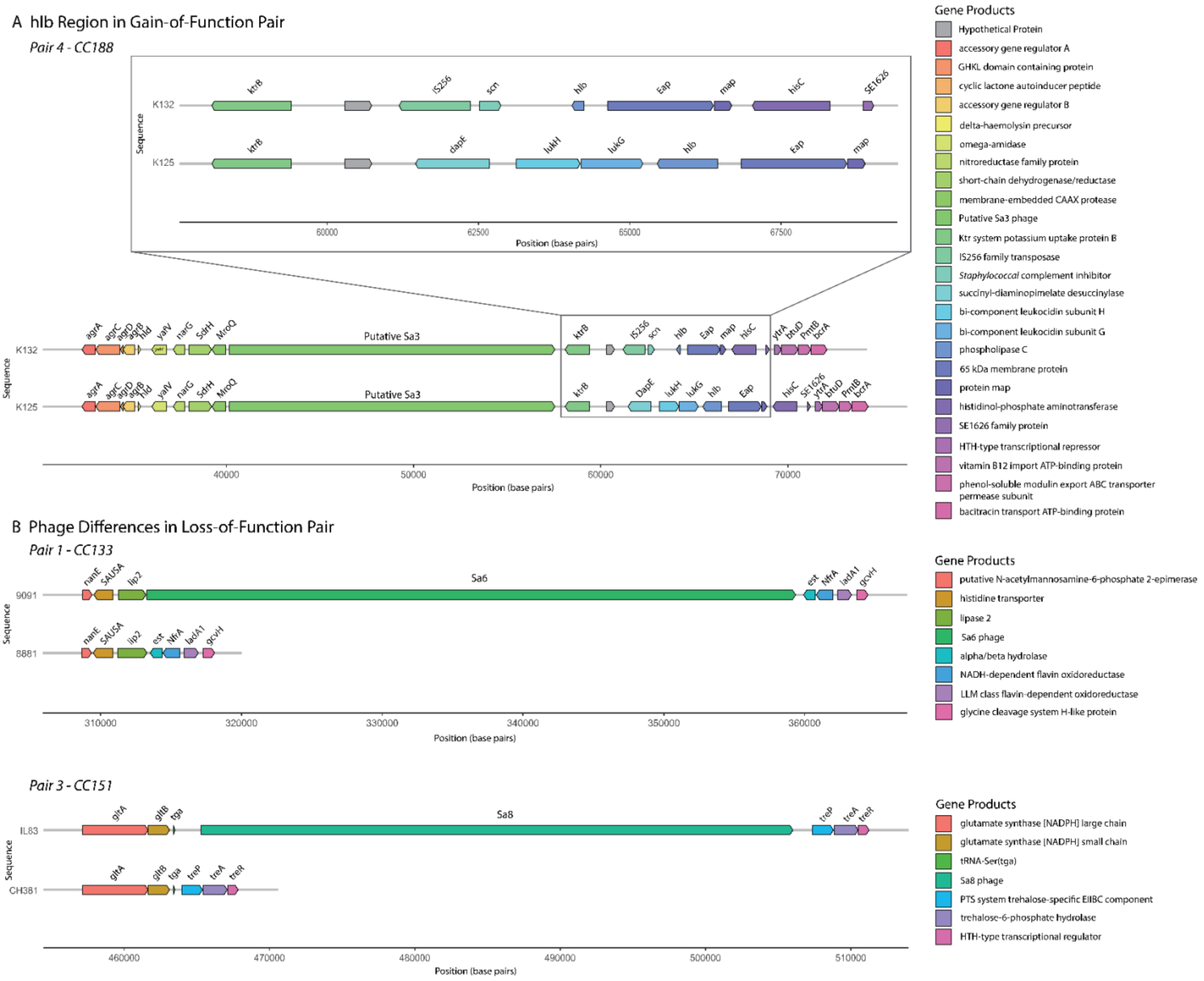

Fig S9. Comparison of selected genomic regions associated with phages that differ between clotting and non-clotting strains in the paired SNP analysis. (A), Gene maps of the *hlb* region in the gain-of-function pair 4 (CC188) showing mobile genetic elements present, the upper panel shows an expanded view of the immediate genes surrounding hlb. K132 represents the clotting strain and K125 represents the non-clotting strain. **(B),** Gene maps of loss-of-function pairs with differences in mobile genetic elements that show the insertion of phages and surrounding genes into the clotting strains in each pair (9091 and IL83) compared to the non-clotting strains (8881 and CH381).

Supplemental Tables

Table S1. The *S. aureus* strains used for genotypic and phenotypic analysis. Includes all strain metadata, clotting phenotype, source of sequence data, and assembly metrics relevant to GWAS inclusion.

Table S2. Genes differentially expressed in TSB. Includes the identity of genes that are differentially expressed by bovine clotting strains in comparison to human or bovine non-clotting strains in TSB media.

Table S3: Genes differentially expressed in milk media. Includes the identity of genes that are differentially expressed by bovine clotting strains in comparison to human or bovine non-clotting strains in milk media.

Table S4: Genomic variation correlated with clotting phenotype. (a) The sequence, significance values, and population distribution frequencies of significant GWAS hits across the dataset and with respect to CCs. (b) A summary of the strains included in each pair in the comparative analysis of gain and loss of function pairs and clades. For clades, variants shared by all members of the clade were compared between a representative isolate (listed in the Clotter/Control Strain column) and an outgroup (listed in the Non-clotter/Comparison Strain column) as a pair, similar to the other pairs. The final column shows the number of SNPs between pairs (after filtering, as described in the Methods). (c) A list of variants identified between gain of function pairs and clades, and corresponding data for each variant. For clades, variants shared by all members of the clade were compared between a representative isolate (listed in the Clotter/Control Strain column) and an outgroup (listed in the Comparison Strain column) as a pair, similar to the other pairs. A 'Filtering Rationale' column is included to highlight variants that were filtered out of analyses and the rationale behind filtering each one. Data for the 'Predicted Effect' and 'Gene Name', and 'Nearest Gene Annotation' are listed as reported by snippy or PROKKA. The 'Name in Supplementary File' column is included to match variants that were not filtered out of the analysis with sequences of the closest gene, as provided in the Supplementary file. (d) A list of variants identified between loss of function pairs, and corresponding data for each variant. A 'Filtering Rationale' column is included to highlight variants that were filtered out of analyses and the rationale behind filtering each one. Data for the 'Predicted Effect' and 'Gene Name', and 'Nearest Gene Annotation' are listed as reported by snippy or PROKKA. The 'Name in Supplementary File' column is included to match variants that were not filtered out of the analysis with sequences of the closes gene, as provided in the Supplementary file. (e) A list of accessory gene clusters (Panaroo) that exhibited the same presence/absence pattern between more than one gain or loss of function pair or clade respectively. (f) A list of large variations in the accessory genome of gain and loss of function pairs and clades that were confirmed by long-read sequencing.

File S1. CDS nucleotide sequences corresponding to the variants identified in pairwise analysis

Table S5. Constructs used in this study

Strain or plasmid	Genotype or description	Reference
pALC2073	Tetracycline-inducible expression vector	https://doi.org/10.1016/j.plasmid.2008.10.001
pCT	Derivative of pALC, C-terminal 6xHis-tag and Strep-tagII	This study
pIMAY-Z	Alelle replacement vector with lacZ blue/white screening	doi: 10.1128/mBio.00308-15
DC10B	DH10B background, Δ*dcm*	doi: 10.1128/mBio.00277-11
AH1919	USA300 LACΔ*aur*Δ*sspAB*Δ*scpA spl*::*erm*	DOI: 10.1128/JB.00369-11
AH919 pALC2073	USA300 LACΔ*aur*Δ*sspAB*Δ*scpA spl*::*erm* empty expression plasmid	This study
AH1919 pCT::*aur*	USA300 LACΔ*aur*Δ*sspAB*Δ*scpA* Δ*spl*::*erm* overexpression *aur*	This study
AR802Δ*aur*	aureolysin deletion	This study
AR802Δ*aur* pCT::*aur*	aureolysin deletion with aureolysin complementation	This study
F40Δ*aur*	aureolysin deletion	This study
F40Δ*aur* pCT::*aur*	aureolysin deletion with aureolysin complementation	This study
E48Δ*aur*	aureolysin deletion	This study
E48Δ*aur* pCT::*aur*	aureolysin deletion with aureolysin complementation	This study
CTH96Δ*aur*	aureolysin deletion	This study
CTH96Δ*aur* pCT::*aur*	aureolysin deletion with aureolysin complementation	This study
NewbouldΔ*aur*	aureolysin deletion	This study
NewbouldΔ*aur* pCT::*aur*	aureolysin deletion with aureolysin complementation	This study
AR802Δ*sspA*	*sspA* serine protease deletion	This study
AR802Δ*sspA* pCT::*sspA*	*sspA* deletion with SspA complementation	This study
F40Δ*sspA*	*sspA* serine protease deletion	This study

CTH96Δ*sspA*	*sspA* serine protease deletion	This study
KVSA-7 pALC2073	empty expression construct	This study
KVSA-7 pCT::*aur*	overexpression of aureolysin	This study
KVSA-7 pCT::*sspA*	overexpression of SspA serine protease	This study
E17 pALC2073	empty expression construct	This study
E17 pCT::*aur*	overexpression of aureolysin	This study
E17 pCT::*sspA*	overexpression of SspA serine protease	This study
E27 pALC2073	empty expression construct	This study
E27 pCT::*aur*	overexpression of aureolysin	This study
55435 pALC2073	empty expression construct	This study
55435 pCT::*aur*	overexpression of aureolysin	This study
83075 pALC2073	empty expression construct	This study
83075 pCT::*aur*	overexpression of aureolysin	This study
H064020492	empty expression construct	This study
H064020492 pCT::*aur*	overexpression of aureolysin	This study
IL117 pALC2073	empty expression construct	This study
IL117 pCT::*aur*	overexpression of aureolysin	This study
IL117 pCT::*sspA*	overexpression of SspA serine protease	This study
CH381 pALC2073::*agr*	*agr* complementation	This study
CH354 pALC2073::*agr*	*agr* complementation	This study
8881 pALC2073::*agr*	*agr* complementation	This study

Table S6. Primers used in this study

Primer Name	Sequence (5'-3'; restriction site underlined, complementary sequence lowercase)
pCT::*aur* F	aataagcttgatggtaccgagctcAAGCTGTTTTTAAGATTTCAG
pCT::*aur* R	aaattgaggatgagaccagaattcCTCCACGCCTACTTC
pCT::*sspA* F	aataagcttgatggtaccgagctcGTAAATAAATTTTTTGGAGGTT
pCT::*ssp*A R	aaattgaggatgagaccagaattcTGCAGCGTCAGG
pALC2073 MCS F	ATACCGCACAGATGCGTAAGG
pALC2073 MCS R	CGATGACTTAGTAAAGCACATCTAA
aur A	cctcactaaagggaacaaaagctgggtaccCATTAGGCATCTGGTTTGTC
aur B	gtttaacattacttcttcttgtttatTTCCTCCTGAAATCTTAAAAACAG
aur C	ctgtttttaagatttcaggaggaaATAAACAAGAAGAAGTAATGTTAAAC
aur D	cgactcactatagggcgaattggagctcTATGAACCATTGATGATTGAACT
aur OUT F	AACGCGATTAAGTATGAT
aur OUT R	CCAGGTGAGGTTTTGAC
sspA A	cctcactaaagggaacaaaagctgggtaccGGACGTCGTGAACTA
sspA B	tactaaatctaaattaagatgaagtttaCATCTAAAAACCTCCAAAAAA
sspA C	ttttttggaggtttttagatgTAAACTTCATCTTAATTTAGATTTAGTA
sspA D	attggagctccaccgcggtggcggccgcTTTAGCACTTTCTTTTTCTTTTACA
sspA OUT F	GCAATCGTTCCAGGCTCATC
sspA OUT R	TCTTCTTGTATCGCTTCGTTTTC
pIMAY-Z MCS F	TACATGTCAAGAATAAACTGCCAAAGC
pIMAY-Z MCS R	AATACCTGTGACGGAAGATCACTTCG
pALC2073::*agr* F	CCCCGGATCCGAAGCGCCCGAAATAATAAT
pALC2073::*agr* R	CCCCGAGCTCTAAAAATTGCGCCATAGGAT
